# An umbrella review of psychological capacity and mental health trajectories across the life course

**DOI:** 10.1038/s44220-026-00592-x

**Published:** 2026-02-26

**Authors:** Darío Moreno-Agostino, Nusrat Khan, Vanessa De Rubeis, Chandni Maria Jacob, Prerna Banati, Ritu Sadana, Matthew Prina

**Affiliations:** 1 Centre for Longitudinal Studies, UCL Social Research Institute, University College London, London, UK; 2 ESRC Centre for Society and Mental Health, King’s College London, London, UK; 3 Population Health Sciences Institute, Medical Sciences Faculty, Newcastle University, Newcastle, UK; 4 Department of Maternal, Newborn, Child, Adolescent Health and Ageing, World Health Organization, Geneva, Switzerland

**Keywords:** Psychology, Risk factors

## Abstract

Understanding population trajectories of psychological capacities can guide interventions to protect and enhance them across the life course. We conducted an umbrella review of systematic reviews examining the trajectories of a wide range of psychological capacity measures. Searches were performed in MEDLINE, EMBASE, PsycINFO, the Cochrane Database of Systematic Reviews and Google Scholar (11 December 2023 and 26 June 2025). Thirty-six reviews synthesizing 1,307 primary studies were included. Here we show that most reviews focused on depression, anxiety and trauma-related symptoms, with stable low-symptom trajectories being most common. Being a girl/woman and socioeconomic disadvantage were frequent risk factors, while social support emerged as protective. We found a comparative lack of reviews focused on less common mental-health conditions, positive outcomes and older adults. Future reviews should engage with a robust quality assessment of the analytical approach used and the (lack of) geographical and sociodemographic diversity in the primary studies included. Similarly, more evidence on the Global South and on minoritized and marginalized groups within populations is needed. The protocol is pre-registered in PROSPERO (CRD42023490490).

## Abstract (web summary)

This analysis conducted an umbrella review of 36 systematic reviews assessing psychological capacity trajectories. The findings indicate prevalent low-symptom patterns, highlighting gender and socioeconomic factors as risks, while emphasizing the need for diverse, quality assessments in future studies.

## Main

Intrinsic capacity is a novel multidimensional concept that aims to represent all the physical and mental capacities that a person can draw on and that, in interaction with the environment, determine a person’s functional ability^1,2^. A key contribution of this concept—and, more widely, of the World Health Organization’s (WHO) healthy aging and life-course frameworks^2,3^—is to shift the focus from deficit-oriented perspectives towards a person-centered approach focused on fostering and maintaining a person’s ‘intrinsic capacity’ across the life course. One of the key subdomains proposed in these frameworks is ‘psychological capacity’. Although only recently proposed, it has so far been largely operationalized using indicators of common mental-health problems, chiefly depressive symptoms^4,5^. There is value in focusing on these conditions as they are among the leading contributors to the global burden of disease^6–8^. However, although essential, this narrow focus^5,9^ risks undermining the proactive, capacity-building philosophy of the broader framework, for example, what contributes to producing health—not only by ensuring the absence of disease. Recent operationalizations have been expanded to also include positive psychological measures such as life satisfaction, optimism and happiness^10^, which represent an important part of human experiences, particularly in the context of increasing longevity, across the life course^11^.

A study of trajectories of different measures of psychological capacity is of particular interest from a life-course perspective^2,3^. Trajectories are based on repeated observations within cohorts of the same individuals over time (longitudinal data), and they thus provide critical insights into how these different measures change as people age. Trajectories may remain stable or change throughout different life stages, and in response to life transitions and challenging experiences. These changes may be systematically similar or different across groups within populations, and some methodological approaches allow detecting such differences with or without pre-specifying what variables define such groups (Box 1 describes some of the key terms in trajectory studies). Hence, the study of psychological capacity trajectories can inform interventions aimed at protecting and fostering this capacity across the life course, as well as help identify which factors lead to inequalities in these and when. This can provide cumulative benefits for older persons through prevention and early intervention.

There is a large body of research using quantitative approaches to study trajectories in outcomes that would fall under the broad psychological capacity domain as conceptualized above. Although valuable, this body of work has become increasingly fragmented, often siloed by specific terminology or outcomes such as depressive symptomatology, or confined to narrow life stages (for example, adolescence or the perinatal period). This fragmentation makes it difficult for researchers and policymakers to gain a holistic, life-course perspective on how psychological capacities evolve. The unique contribution of this umbrella review is its synthesis of this broad and disparate evidence base. By bringing together all existing reviews under a single, overarching conceptualization of psychological capacity, inclusive of both positive and negative factors, our review offers a high-level integrated overview of the field^12,13^.

This approach offers a more holistic view of key findings, similarities and differences across otherwise typically more fragmented concepts, and helps pinpoint critical life stages and periods at which psychological capacities may shift, which can be of great relevance from a policy perspective. This analysis can also help identify potential gaps in the existing literature (for example, underrepresented populations, psychological capacity operationalizations, methodological approaches, life stages and geographical areas), as well as evaluate the quality and consistency of existing reviews, providing valuable insights for future research.

The aim of this umbrella review is to synthesize systematic reviews on the trajectories of psychological capacity across the life course to identify gaps and patterns in the evidence.

Box 1 Relevant concepts in trajectory studies The present umbrella review is concerned with systematic reviews of trajectory studies. Trajectories represent the change in a particular outcome over a period of time within the same units (because we are concerned with trajectories of psychological capacities, individuals are the unit of interest). Trajectories require the analysis of longitudinal data—more specifically, the use of repeated measures within cohorts of the same individuals over time. Although a straight trajectory could be drawn between any two repeated observations, the resulting trajectory would simply represent the change or difference between the two. Although this provides an accurate estimate of the difference, such an estimate may not be generalizable to other, even similar, scenarios, as it may strongly depend on the characteristics of the data used (a phenomenon called ‘overfit’). With three or more observations, a straight trajectory can be used to summarize the underlying change in the repeated observations in a more generalizable and parsimonious way.

Assuming that the trajectory is a straight line, such a line can be summarized simply using two key quantities, regardless of how many repeated observations are being used: the intercept (usually, the starting point^1^) and the slope (the amount of change that is expected over a certain amount of time^2^). These two quantities are usually assumed to be either the same for every individual (fixed, less flexible but more parsimonious) or subject to certain variation (random, more flexible but less parsimonious). In the latter case, estimates of the variance of the intercept and/or the slope can be obtained, representing whether and by how much the starting point and the change over time vary across individuals.

Growth curve models (either multilevel or latent) are flexible methodological approaches that can be used at the population level to estimate these different quantities (see ref. ^79^ for an overview of the methodological approach). Sometimes, these quantities may vary in systematic ways across individuals. Variable-centered approaches such as conditional and multiple group growth curve models may be used to explore whether specific variables (or combinations of variables) are associated with different levels in the intercept, slope(s) or both, as well as with their degree of variability. Person-centered approaches such as latent class growth analysis (LCGA) or the more flexible growth mixture model (GMM) are often used to identify subgroups within the population under study with different trajectories, without pre-specifying the grouping variables. Subsequent analyses can then be performed to explore the relationship between different variables and the probability of being in the different resulting trajectories^80^.

^1^The intercept most often, but not always, represents the starting point. However, to simplify the exposition, we will refer to it as the ‘starting point’ of the trajectory.

^2^If this amount of change is always the same, a linear slope may be sufficient to appropriately characterize the trajectory. However, additional slopes or different analytical approaches may be needed if the amount (and even direction) of change varies over time (examples of such approaches are provided in refs. ^79^ and ^81^).

## Results

### Study selection

**Fig. 1:**
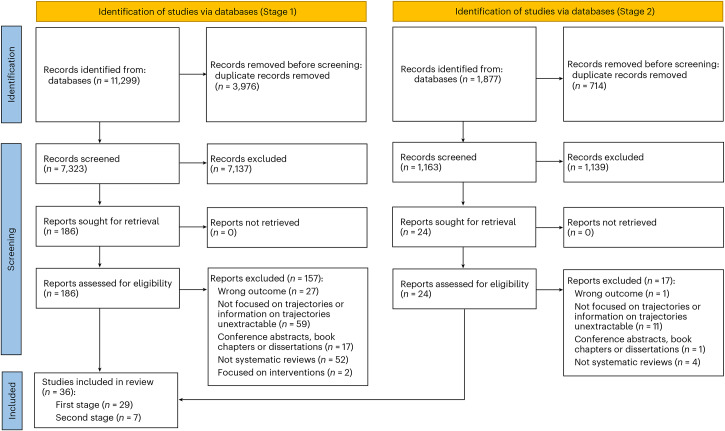
PRISMA flowchart. Figure adapted from ref. ^76^ under a Creative Commons license CC BY 4.0.

The searches (Methods) yielded 13,176 potentially eligible articles (11,299 and 1,877 from the first and second stages, respectively). After removal of duplicates, 8,486 studies (7,323 and 1,163, respectively) remained. Title and abstract screening resulted in 210 studies included for full text assessment (186 and 24, respectively). After full text screening, a total of 36 reviews were selected for inclusion in our review, 29 from the first stage and seven from the second (Fig. 1). The agreement (inter-rater reliability) between reviewers was 99.0% (Cohen’s *κ* = 0.76) and 99.8% (Cohen’s *κ* = 0.87) in the first and second screening stages, respectively. A list of papers excluded at full text assessment, with the reason for exclusion, is provided in Supplementary Table 1.

### Characteristics of the included reviews

This Analysis paper includes evidence from 36 systematic reviews and 1,307 primary research articles (Table 1). The publication year for the reviews spanned from 2009 to 2025, with over half of the articles published after 2020. The total number of participants across 36 reviews was 2,491,934 (range 1,002–512,473), which does not account for potential overlap of primary studies. This total excludes one review that did not report sample sizes. Most studies (*n* = 28) included adults and assessed multiple life stages^14–41^, with fewer focusing specifically on early life stages (*n* = 7)^42–48^ and only one concentrating on older adults alone^49^. The majority of systematic reviews did not have a focus on populations with specific health characteristics or conditions (Table 1). Trajectories of general mental health and common mental disorders were reviewed across 24 reviews; 16 covered trauma and stressor-related trajectories, three covered subjective wellbeing and positive outcomes, one suicidality and eating disorders, and one schizophrenia symptoms. Because all these outcomes can be conceptualized as mental ill-health and wellbeing, or more broadly as mental health, we use the term ‘mental health’ from here onwards. Different conceptualizations of trajectories were used across the different reviews, with two-thirds (*n* = 23; 64%) of the reviews utilizing a person-centered approach (Box 1). Across the reviews, many countries were represented. However, an analysis of the geographical distribution of the primary studies within the included reviews reveals a substantial concentration of research in high-income, English-speaking countries. The United States was the most represented country by a substantial margin, accounting for 277 studies across the reviews. Following the United States, the most frequent locations for research were China (115 studies), Australia (64), Canada (55), the Netherlands (52) and the United Kingdom (41). Although research from other parts of Europe and Asia was also present, there was a notable underrepresentation of studies from low- and middle-income countries, particularly from South America, Africa and Western Asia.

**Table 1:** Characteristics of the reviews

Authors	Years covered	No. of included studies^a^	Population of interest		Main outcome(s) of interest				Life stage				Focus on person-centered approaches	Key findings
			Category	Sub-category	Common mental-health symptoms	Trauma and stressor-related outcomes	Wellbeing and positive outcomes	Other	Infancy/childhood	Adolescence	Adulthood	Older adulthood		
Baron et al.^14^	Inception–2015	11	Defined by experience/event	Perinatal	Perinatal depressive symptoms					X	X		Yes	A low-stable symptom trajectory was universally identified; stable moderate–high and transient (increasing/decreasing/episodic) trajectories were also common. No systematic predictors were found across studies.
Bonde et al.^15^	Inception–2021	34	Defined by experience/event	Adults		PTSD/PTSS					X	X	Yes	Delayed PTSD trajectories were identified, characterized by initially elevated symptoms that worsened over time relative to resilient groups; findings showed high variation and methodological limitations.
Cohen-Mansfield et al.^49^	2003–2017	50 (7)	General population	Older adults			Life satisfaction/ positive and negative affect					X	No	Subjective wellbeing trajectories were stable throughout adulthood, with a terminal decline beginning ~3–5 years before death.
Curran et al.^36^	Inception–2023	14	Living with specific health condition	Adults with cancer	Depressive symptoms/ anxiety symptoms/internalizing and externalizing symptoms	PTSD/PTSS					X	X	No	Found high heterogeneity in depression/anxiety trajectories, with up to 20% experiencing persistent symptoms. Physical functioning was the most consistent predictor of trajectory membership.
Danielsson et al.^42^	1990–2021	49	Living with specific health condition	Children with neurodevelopmental disabilities	Depressive symptoms/anxiety symptoms/internalizing and externalizing symptoms				X	X			No	Trajectories of depression/anxiety were largely stable, with almost half changing by less than 10% between first and last assessments.
De Sousa et al.^37^	2013–2023	37	General population	Adults	Depressive symptoms						X	X	No	Depression trajectories in adults were highly heterogeneous. Social support and physical activity were protective, while socioeconomic disadvantage and female gender were risk factors.
Galatzer-Levy et al.^16^	Inception–2016	54	Defined by experience/event	Trauma and disasters (any)		Resilience^a^			X	X	X	X	Yes	Four core trajectories identified: resilience (most prevalent at 65.7%), recovery, chronic and delayed onset.
Gendre et al.^38^	1996–2023	18	Defined by experience/event	Trauma and disasters (any)		PTG			X	X	X	X	Yes	Identified both level-based (stable, increasing, decreasing) and feature-based (constructive, illusory, struggling) PTG trajectories. Higher/constructive trajectories were predicted by active coping and social support.
Gilbey et al.^17^	Inception–2018	10	General population	Same-gender attracted youth (<25)	Depressive symptoms			Suicidality/eating disorder symptoms		X	X		Partly	Mental-health disparities for same-gender attracted youth were present from early ages (10–25 years), with risk for depression and suicidality peaking in late adolescence.
Habtewold et al.^18^	2008–2019	53 (19)	Living with specific health condition	Adults with schizophrenia, relatives and controls				Schizophrenia symptoms/ cognitive deficits		X	X		Yes	Four stable, deteriorating, relapsing or improving symptom trajectories were common in schizophrenia.
Infurna et al.^19^	Inception–2018	77	Defined by experience/event	Trauma and disasters (adults)	Depressive symptoms/ anxiety symptoms/psychological distress	PTSD/PTSS	Life satisfaction/ positive and negative affect				X	X	Yes	Studies that did not make certain widespread methodological assumptions (homogeneity of variances across trajectories and no slope variance within trajectories) found that low-stable symptom trajectories were among the least prevalent.
Kuo et al.^20^	Inception–2015	6	Defined by experience/event	Caregiving and bereavement	Depressive symptoms						X	X	Yes	Most bereaved family members recovered to pre-loss depression levels within one year, but a persistent chronic group (9.9%) remained.
Lai et al.^43^	Inception–2016	8	Defined by experience/event	Trauma and disasters (children)		PTSD/PTSS			X	X			Partly	Identified resilience (most prevalent), recovery and chronic trajectories in children post-disaster. Female gender, trauma exposure and low social support predicted chronic trajectories.
Lemyre et al.^21^	2019–2022	19 (15)	Defined by experience/event	University students during the COVID-19 pandemic			Life satisfaction/ positive and negative affect			X	X		No	Wellbeing in university students decreased at the start of the pandemic, followed by mixed trajectories. Higher SES, social connection and healthy habits were protective.
Magan et al.^22^	1994–2021	16	Defined by experience/event	Caregiving and bereavement	Depressive symptoms						X	X	No	Following patient death, caregivers’ depressive symptoms remained elevated and did not recover to the levels of non-caregiving controls.
Majdandzic et al.^39^	1996–2023	22	Defined by experience/event	Trauma and disasters (adults)		PTG					X	X	Yes	PTG trajectories were mostly stable or increasing, though decreases were also noted. Active-adaptive coping was consistently linked to higher PTG, whereas the role of distress was inconsistent.
Musliner et al.^23^	1999–2015	25	General population	Any	Depressive symptoms				X	X	X	X	Yes	A large majority of participants followed a low-stable symptom trajectory; a minority (<10%) experienced persistent symptoms, predicted by female gender and lower SES.
Nandi et al.^24^	1995–2008	46 (29)	General population	Any	Depressive symptoms/ anxiety symptoms	PTSD/PTSS			X	X	X	X	Yes	Identified heterogeneous trajectories for depression, anxiety, hyperactivity and PTSD. Female gender and lower SES consistently predicted more adverse trajectories.
Newnham et al.^25^	2000–2022	205	Defined by experience/event	Trauma and disasters (any)	Depressive symptoms/ anxiety symptoms	PTSD/PTSS			X	X	X	X	No	Post-disaster PTSD symptoms improved over time, but depression and anxiety remained elevated, especially in younger age groups.
Oppo et al.^40^	Inception–2024	10	Defined by experience/event	Trauma and disasters (adults)		PTSD/PTSS					X	X	Yes	Four core PTSS trajectories identified after collective violence: low-stable (most prevalent at 58%), decreasing (13%), delayed-worsening (8%) and high-stable (7%).
Pavlacic et al.^26^	Inception–2019	27	Defined by experience/event	Veterans and service members		PTSD/PTSS					X	X	Yes	Resilience was the most common trajectory (73.4%) for veterans and service members, with the remaining group showing heterogeneous symptomatic pathways.
Portogallo et al.^48^	2002–2023	12	General population	Children and adolescents	Depressive symptoms				X	X	X		Yes	Identified low (70.3%), moderate, high, increasing and decreasing symptom trajectories in adolescents. Higher symptom trajectories predicted adult depression.
Santiago et al.^27^	1998–2010	58	Defined by experience/event	Trauma and disasters (any)		PTSD/PTSS					X	X	No	PTSD prevalence decreased over 12 months, but this was driven by non-intentional trauma. For intentional trauma survivors, PTSD prevalence steadily increased.
Santos et al.^28^	Inception–2016	23 (22)	Defined by experience/event	Perinatal	Perinatal depressive symptoms					X	X		Yes	Identified at least three maternal depression trajectories (low, medium, high-chronic). Children of mothers in high-symptom trajectories had worse health outcomes.
Schafer et al.^29^	2000–2001	28	Defined by experience/event	Trauma and disasters (any)	Depressive symptoms/ anxiety symptoms/psychological distress	PTSD/PTSS					X	X	Yes	Pandemic distress trajectories mirrored responses to individual stressors, but with fewer recovery trajectories. Older adults showed more resilient responses.
Schubert et al.^30^	Inception–2015	47	General population	Adolescence and early youth	Depressive symptoms					X	X		Partly	A large majority of youth (60–80%) followed a low-stable depressive symptom trajectory, which peaked around age 17. Parental support and higher SES were protective.
Scott et al.^31^	Inception–2023	67	Living with specific health condition	Chronic conditions	Depressive symptoms/anxiety symptoms						X	X	Yes	Most individuals with chronic disease followed a non-clinical (low-stable) trajectory for depression (69%) and anxiety (73.4%); a smaller group (~12–14%) remained in the clinical range.
Shore et al.^44^	1994–2016	20	General population	Children and adolescents	Depressive symptoms				X	X	X		Yes	Identified low (56%), moderate (26%) and high (12%) depressive symptom trajectories. Female gender, low SES and stress reactivity predicted high-symptom groups.
Sun et al.^32^	2012–2022	24 (18)	Defined by experience/event	Caregiving and bereavement	Depressive symptoms	PTSD/PTSS				X	X	X	No	Caregiver resilience showed trajectories of stability, growth and decline, but with notable inconsistency in measurement and predictors across studies.
Tafolla et al.^41^	2000–2023	13 (5)	Living with specific health condition	Autism spectrum disorder	Depressive symptoms/ anxiety symptoms				X	X	X		No	Anxiety and depression trajectories in individuals with ASD were heterogeneous. Adaptive skills and peer relationships were key predictors of symptom change.
Tarren-Sweeney et al.^45^	NR–2018	14	Defined by experience/event	Children in family-based out-of-home care	Depressive symptoms/ anxiety symptoms/ internalizing and externalizing symptoms				X	X	X		No	Children in out-of-home care showed mixed mental-health trajectories, with sizable proportions both improving and deteriorating over time.
Van Den Kieboom et al.^33^	2014–2019	11	Defined by experience/event	Caregiving and bereavement	Psychological distress			Caregiver burden			X	X	No	Caregiver burden most commonly increased over time (up to 36 months). Burden only decreased after the care recipient was admitted to a long-term facility.
Vanwetswinkel et al.^34^	Inception–2021	33	Defined by experience/event	Perinatal	Perinatal depressive symptoms						X		Yes	Most perinatal women (up to 92%) were in low-symptom trajectories, with a small (<15%) high-symptom group. History of depression was the strongest predictor of high symptoms.
Wang et al.^46^	Inception–2011	85 (25)	Defined by experience/event	Trauma and disasters (children)	Depressive symptoms/ anxiety symptoms	PTSD/PTSS			X	X			No	Most children showed declining PTSD symptoms post-disaster, though in some studies symptoms remained stable or increased over time.
Waqas et al.^35^	Inception–2022	66 (53)	Defined by experience/event	Perinatal	Perinatal depressive symptoms						X		Yes	Perinatal women facing sociocultural stressors showed more severe and persistent depressive symptom trajectories, which were linked to poor child outcomes.
Witt et al.^47^	Inception–2021	15	Defined by experience/event	Trauma and disasters (children)		PTSD/PTSS			X	X			Yes	Identified resilience, recovery and chronic trajectories post-disaster, with dynamic changes in the first year. Female gender and low social support predicted worse outcomes.

^a^The figures in parentheses represent the number of longitudinal studies within systematic reviews that extracted information from studies with non-longitudinal designs. ASD, autism spectrum disorder; NR, not reported; PTG, post-traumatic growth; PTSD, post-traumatic stress disorder; PTSS, post-traumatic stress symptoms; SES, socioeconomic status.

### Quality of included reviews

The methodological quality of the included systematic reviews, as assessed by the AMSTAR-2 tool, was highly variable (Table 2). Nearly all reviews clearly defined their patient/population, intervention, comparison and outcome (PICO) components and provided adequate descriptive characteristics of their included studies. However, we identified several systematic and critical weaknesses across most reviews. Most notably, a high proportion of reviews lacked a pre-registered protocol (67%) and failed to provide a list of excluded studies (83%), increasing the risk of reporting bias and reducing transparency. Furthermore, key processes to minimize error and bias, such as performing data extraction in duplicate, were frequently omitted (72%). Over half of the included reviews (53%) did not assess the methodological quality or risk of bias of their primary studies and, among the reviews that did conduct a quality assessment, a majority (59%) failed to incorporate these risk-of-bias findings into the interpretation and discussion of their results.

**Table 2:** AMSTAR-2 ratings for included reviews

Author	1. PICO components	2. Protocol	3. Study design explanation	4. Comprehensive search strategy	5. Duplicate study selection	6. Duplicate data extraction	7. Details of excluded studies	8. Description of included studies	9. Risk of bias assessment	10. Report on funding sources	11. Appropriate meta-analytical methods	12. Risk of bias impact of results	13. Account for risk of bias in discussion	14. Account of heterogeneity in discussion	15. Publication bias investigation	16. Report of conflict of interest
Baron et al.^14^	Yes	Yes	Yes	Yes	Yes	No	No	Yes	Yes	No	NA	NA	Yes	Yes	NA	Yes
Bonde et al.^15^	Yes	Yes	Yes	Yes	Yes	Yes	Yes	Yes	Yes	No	NA	NA	No	Yes	NA	Yes
Cohen-Mansfield et al.^49^	Yes	No	Yes	Yes^a^	No	No	No	Yes^a^	No	No	NA	NA	No	No	NA	Yes
Curran et al.^36^	Yes	No	Yes	Yes	Yes	Yes	No	Yes	Yes	No	NA	NA	Yes	Yes	NA	Yes
Danielsson et al.^42^	Yes	Yes	Yes	Yes	Yes	Yes	No	Yes	Yes	No	NA	NA	Yes	Yes	NA	Yes
De Sousa et al.^37^	Yes	No	Yes	No	Yes	No	No	Yes^a^	No	No	NA	NA	No	Yes	NA	Yes
Galatzer-Levy et al.^16^	Yes	No	Yes	Yes	No	No	No	Yes	No	No	Yes	No	No	Yes	Yes	Yes
Gendre et al.^38^	Yes	No	Yes	Yes	No	No	Yes	Yes	Yes	No	NA	NA	No	No	NA	Yes
Gilbey et al.^17^	Yes	Yes	Yes	Yes	No	No	No	Yes	Yes	No	NA	NA	Yes	Yes	NA	Yes
Habtewold et al.^18^	Yes	Yes	Yes	Yes	Yes	Yes	No	Yes	No	No	NA	NA	No	Yes	NA	Yes
Infurna et al.^19^	Yes	No	Yes	Yes	No	No	No	Yes	No	No	NA	NA	Yes	Yes	NA	No
Kuo et al.^20^	Yes	No	Yes	Yes	Yes	Yes	No	Yes	Yes	No	NA	NA	Yes	Yes	NA	Yes
Lai et al.^43^	Yes	No	No	Yes	No	No	No	Yes	No	No	NA	NA	No	Yes	NA	No
Lemyre et al.^21^	Yes	Yes	Yes	Yes	Yes	Yes	No	Yes	Yes	No	NA	NA	Yes	Yes	NA	Yes
Magan et al.^22^	Yes	No	Yes	Yes	No	No	No	Yes	Yes	No	NA	NA	Yes	No	NA	Yes
Majdandzic et al.^39^	No	Yes	No	No	Yes	No	No	Yes	Yes^a^	No	NA	NA	No	Yes	NA	Yes
Musliner et al.^23^	Yes	No	Yes	Yes^a^	No	No	No	Yes	No	No	NA	NA	No	No	NA	No
Nandi et al.^24^	Yes	No	Yes	No	No	No	No	Yes	No	No	NA	NA	No	No	NA	Yes
Newnham et al.^25^	Yes	Yes	Yes	Yes	No	No	Yes	Yes	No	No	Yes	Yes	Yes	Yes	No	Yes
Oppo et al.^40^	Yes	No	Yes	Yes^a^	Yes	No	No	Yes	Yes^a^	No	Yes	No	No	Yes	Yes	No
Pavlacic et al.^26^	Yes	No	Yes	No	Yes	Yes	No	No	No	No	NA	NA	No	No	NA	No
Portogallo et al.^48^	Yes	No	Yes	Yes	Yes	No	No	Yes	Yes	No	Yes	No	No	Yes	No	Yes
Santiago et al.^27^	No	No	No	Yes	No	No	No	No	No	No	NA	NA	No	No	NA	Yes
Santos et al.^28^	Yes	No	Yes	Yes	Yes	No	No	Yes	No	No	NA	NA	No	No	NA	Yes
Schafer et al.^29^	Yes	No	Yes	Yes	Yes	Yes	No	Yes	No	No	Yes	No	No	Yes	NA	Yes
Schubert et al.^30^	Yes	No	Yes	Yes^a^	No	No	No	Yes	No	No	NA	NA	No	Yes	NA	Yes
Scott et al.^31^	Yes	Yes	Yes	Yes^a^	Yes	Yes	No	Yes	Yes	No	Yes	No	No	Yes	No	Yes
Shore et al.^44^	Yes	No	Yes	Yes^a^	No	No	No	Yes	Yes^a^	No	Yes	No	No	Yes	No	Yes
Sun et al.^32^	Yes	Yes	Yes	Yes^a^	Yes	Yes	No	Yes	No	No	NA	NA	No	Yes	NA	Yes
Tafolla et al.^41^	Yes	Yes	Yes	Yes	No	No	No	No	No	No	NA	NA	No	No	NA	Yes
Tarren-Sweeney et al.^45^	Yes	No	Yes	Yes	No	No	Yes	Yes	No	No	NA	NA	No	Yes	NA	Yes
Van Den Kieboom et al.^33^	Yes	No	Yes	Yes^a^	No	No	No	Yes	Yes	No	NA	NA	No	Yes	NA	Yes
Vanwetswinkel et al.^34^	Yes	No	Yes	Yes^a^	No	No	No	Yes	No	No	NA	NA	No	Yes	NA	Yes
Wang et al.^46^	Yes	No	Yes	Yes	No	No	No	Yes	Yes	No	NA	NA	No	No	NA	Yes
Waqas et al.^35^	Yes	Yes	Yes	Yes	Yes	No	No	Yes	Yes	No	NA	NA	Yes	Yes	NA	Yes
Witt et al.^47^	Yes	No	No	Yes	Yes	No	No	Yes	No	No	NA	NA	No	No	NA	Yes

Yes^a^, partial yes; NA, not available. The full question list is available from ref.^52^.

### Trajectories of psychological capacity in populations defined by experiences or events

Twenty-three reviews (*n* = 23, 64%) assessed trajectories defined by experiences or events^14–16,19–22,25–29,32–35,38–40,43,45–47^. This could be broadly subcategorized into studies of trauma and disasters (*n* = 11)^15,16,19,25,27,38–40,43,46,47^, women in the perinatal period (*n* = 4)^14,28,34,35^, caregiving and bereavement (*n* = 4)^20,22,32,33^ and other specific contexts (*n* = 4), which included the COVID-19 pandemic^21,29^, children in family-based out-of-home care^45^, and veterans and service members^26^.

### Trauma and disasters

**Fig. 2:**
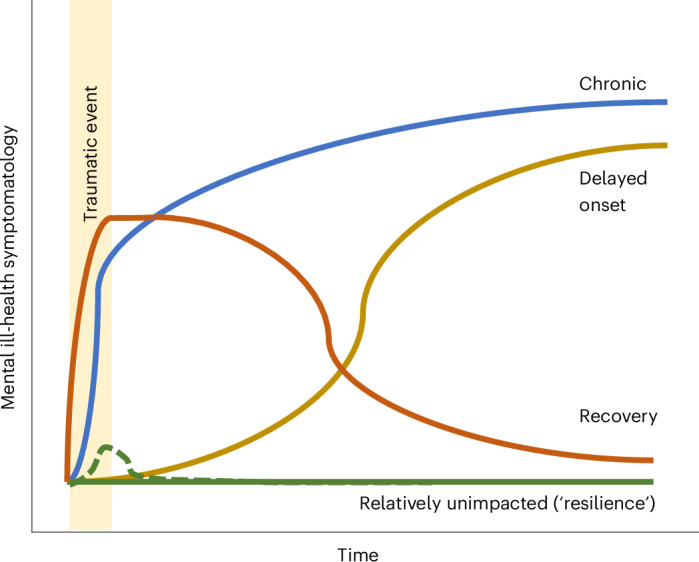
Examples of types of trajectory frequently found in studies using person-centered approaches. Note that this figure is based on the types of trajectory frequently reported in the included systematic reviews when extracting evidence from studies that used person-centered approaches, and it does not aim to be exhaustive. The dashed line represents a different typology of the relatively unimpacted (‘resilience’) trajectory type, capturing an initial, although reduced, impact with a rapid return to pre-existing low levels of symptomatology.

Most reviews identified four distinct trajectories: relatively unimpacted (sometimes named ‘resilient’), recovery, chronic and delayed-onset trajectories (Fig. 2). Some reviews identified other trajectories across primary papers, but these were heterogeneous in patterns and name and were therefore not synthesized. Across primary studies, the ‘resilient’ trajectory was the most common, followed by recovery, chronic and delayed onset trajectories^16^. There was consistent evidence to suggest that individuals with delayed post-traumatic stress disorder (PTSD) symptoms had higher symptoms (30%) during the first months compared to people in the low-stable trajectory, with few individuals experiencing a completely asymptomatic delay^15^. This general model held true for various traumatic events and populations, including natural disasters, veterans and community-based adults.

However, a review identified that civilians initially exhibit higher PTSD symptom trajectories with a flatter slope compared to military personnel^15^. The prevalence of these trajectories also varied systematically by population. The ‘resilient’ trajectory was highest in military populations and lowest among children^16^. For example, Pavlacic and colleagues^26^ identified that among veterans and service members, an average of 73.4% of participants across primary studies reported a ‘resilient’ trajectory, with the remainder experiencing trajectories characterized by different patterns of PTSD/post-traumatic stress symptoms (PTSS). Oppo and colleagues^40^, on the other hand, focused on populations exposed to collective violence, such as war, terror and displacement. Their work also identified similar trajectories, but while confirming resilience as the most common outcome, they found a lower prevalence of the recovery trajectory compared to reviews of more generalized trauma (13%), suggesting that the prolonged and cumulative nature of stressors in the contexts of war and forced displacement could make recovery a more challenging process^15^.

As opposed to PTSD, symptoms of anxiety and depression were reported to stay elevated years after disasters and pandemics, with younger age groups reporting higher symptoms across all time points^25^. It was also observed that the percentage of individuals within a specific trajectory remained relatively stable across studies and was not particularly influenced by the objective severity of the event. However, another review highlighted that the severity of the event could still impact the intensity and magnitude of the trajectories experienced^16^.

In children, a similar set of trajectories was identified^43,46,47^, with a common pattern of dynamic development in the first 6–12 months followed by greater stability, and with factors including female gender, high trauma exposure and low social support predicting worse outcomes.

In addition to the trajectories of distress, two systematic reviews specifically synthesized evidence on the phenomenon of post-traumatic growth (that is, positive change after experiencing a traumatic event), revealing a complex and heterogeneous process^38,39^. These reviews consistently found multiple distinct trajectories, most commonly described as stable (at low, moderate or high levels), increasing or decreasing patterns of growth over time. A critical theme in both reviews was the distinction between ‘constructive’ growth, reflecting genuine positive change, and ‘illusory’ growth, which may act as a coping mechanism without long-term functional improvement. Membership in more adaptive or ‘constructive’ trajectories was consistently linked to the use of active-adaptive coping strategies. The role of distress was more complex, with evidence suggesting it can coexist with growth or that moderate levels of distress—but not excessively high or low levels—are most predictive of positive change. These findings underscore that post-traumatic growth is not a uniform outcome, but a dynamic process with multiple possible pathways, shaped by individual coping styles and the struggle with post-event distress.

### Perinatal depression

Four systematic reviews on perinatal depressive symptoms^14,28,34,35^ identified a consistent pattern analogous to the trauma literature: a large low-stable group and a smaller, persistent high-stable group. These reviews also found non-stable trajectories reflecting sudden drops, episodic increases or constant increases in symptoms. Factors consistently associated with worse-off trajectories included lower income/education, lack of social support, minority status, a history of mental-health problems and younger maternal age. Two reviews also highlighted that worse maternal depression trajectories were linked to adverse physical, cognitive and mental-health outcomes in their children^28,35^.

### Caregiving and bereavement

In the context of caregiving and bereavement, trajectories of decreasing depressive symptoms after the loss of a family member were common^20,22^. However, substantial heterogeneity was noted, with notable minorities experiencing prolonged distress, described as ‘chronic grief’ or ‘chronic depression’^20^. Van den Kieboom and colleagues^33^ found that, among informal caregivers of people with dementia, most evidence pointed at increasing caregiver burden over time, which some studies reported only receded upon admission of the care recipient into a care facility. Social support was identified as a key protective factor for caregivers facing healthcare challenges^32^.

### Other specific contexts

Two reviews focusing on the COVID-19 pandemic also identified patterns of resilience and distress^21,29^. Schäfer and colleagues^29^, in an outcome-wide review, found that a large low-stable (or high-stable for wellbeing) trajectory was most prevalent, followed by recovery, delayed negative response and stable-high symptom trajectories. Social support and internal locus of control were associated with more adaptive pathways. Lemyre and colleagues^21^ focused on university students and noted a general reduction in wellbeing at the pandemic’s onset, followed by mixed trajectories thereafter.

Finally, a review focusing on children who experienced maltreatment and grew up in family-based out-of-home care (that is, care provided by non-biological families such as foster or kinship carers) found mixed results regarding their long-term mental health, highlighting a critical need for more evidence on the specific factors that influence their trajectories^45^.

### Trajectories of psychological capacity in the general population

Eight (*n* = 8; 22%) of the included reviews synthesized evidence on changes in psychological capacity over time, without explicitly focusing on a trigger event^17,23,24,30,37,44,48,49^. These reviews examined developmental or aging trajectories in specific outcomes including depressive symptomatology^23,30,37,44,48^ or subjective wellbeing^49^, as well as broader outcome operationalizations either in multiple^24^ or specific populations^17^.

Across five reviews focusing on depressive symptomatology in the general population, a highly consistent pattern of heterogeneity emerged. The most common finding was a large ‘low-stable’ trajectory, comprising the majority of individuals. This was observed across the lifespan, from studies of children and adolescents by Shore et al.^44^ and Portogallo et al.^48^ to young people by Schubert et al.^30^ (where it comprised 60–80% of the reference populations), and in long-term studies including adults and older adults by de Sousa et al.^37^ and Musliner and colleagues^23^. Alongside this, a smaller but persistent ‘high-stable’ or chronic group was consistently identified, typically representing 5–12% of the reference population. Several reviews also found evidence of more dynamic patterns, identifying trajectories of moderate, increasing and decreasing symptoms. Crucially, this heterogeneity was shown to have impacts across life stages, with Portogallo and colleagues^48^ reporting that higher symptom trajectories during adolescence were predictive of a formal depression diagnosis or ‘caseness’ in young adulthood.

A particularly robust finding, confirmed in reviews by Musliner et al.^23^, Schubert et al.^30^, Shore et al.^44^ and de Sousa et al.^37^, was the consistent association of female gender and lower socioeconomic position with worse-off, higher symptom trajectories. Conversely, protective factors such as parental/familial support, social support from peers, and physical activity were identified as predicting membership in more adaptive, low-symptom pathways.

Gilbey and colleagues^17^ found evidence that adolescence may be the period of greatest risk of suicidal ideation. However, they reported inequalities in depressive symptoms and suicidality between same-gender attracted and heterosexual youth from the earliest observations at ages 10–11 and 16 years, respectively, with those inequalities persisting into adulthood and never completely dissipating.

Nandi and colleagues^24^ found that most available evidence on heterogeneous trajectories (and typologies) in different mental-health outcomes within populations from different age groups had focused on depressive symptomatology and had been conducted in children, adolescents and young adults. They also found that being a girl/woman, being in a more disadvantaged socioeconomic situation, having lower social support, and experiencing more stressful life events were consistently associated with worse-off trajectories across studies and outcomes.

Finally, in their review on terminal decline (that is, mortality-related deterioration that ends in death), and the only review that focused on older adults, Cohen-Mansfield and colleagues^49^ found that, while life satisfaction was relatively stable throughout adulthood, declines could be observed at ~3–5 years before death.

### Trajectories of psychological capacity in populations with specific health conditions

Five studies^18,31,36,41,42^ assessed psychological capacity in populations living with specific health conditions, consistently finding patterns of both stability and distress.

The most common finding across diverse conditions was the presence of a large majority of individuals in a ‘low-stable’ or resilient trajectory. This was clearly quantified by Scott and colleagues^31^, who found that 69% of individuals with chronic diseases maintained low depressive symptoms and 73% maintained low anxiety. This pattern was echoed in the review by Curran et al.^36^ on adults with cancer, where ‘most’ individuals maintained stable mental health over time, but ~10% experienced persistent negative outcomes, often predicted by comorbidities or lower socioeconomic position. Alongside this resilient group, a smaller but persistent ‘high-stable’ or chronic trajectory was also consistently identified. Scott and colleagues^31^ reported this group comprised 12–14% of their population, a figure remarkably similar to the 10% who experienced persistent negative outcomes in the review by Curran and others^36^.

This heterogeneity was also evident for neurodevelopmental conditions. Danielsson and colleagues^42^, focusing on children with conditions including attention deficit hyperactivity disorder (ADHD) and autism, reported that although stable outcomes were common, there was a tendency for internalizing symptoms to increase over time as externalizing symptoms decreased. Further highlighting this variability, Tafolla and Lord^41^ also identified diverse trajectories of anxiety and depression, specifically among people with autism.

In a review mostly focused on patients living with schizophrenia spectrum disorders, Habtewold and colleagues^18^ found that heterogeneity in schizophrenia symptoms was the norm, with an average of four distinct trajectories (ranging from 2–5) being the most replicated finding for positive, negative and cognitive symptoms.

All five reviews pointed to different combinations of common predictors of more adverse trajectories, including sociodemographic factors and lower socioeconomic position, lack of social support, and clinical factors such as condition severity and/or comorbidities^18,31,36,41,42^.

## Discussion

This is the first umbrella review to provide a comprehensive overview of systematic reviews on trajectories of positive and negative psychological outcomes across the whole life course. In this umbrella review we identified a large but fragmented literature on trajectories of psychological capacities encompassing 36 systematic reviews, including over 1,300 original papers and almost 2.5 million individuals. Most of the evidence that was assessed focused on mental-health problems, predominantly depressive, anxiety and post-traumatic stress symptomatology, after a specific traumatic or life event, rather than on positive outcomes, such as life satisfaction or positive affect. The majority of the systematic reviews focused on primary studies that used person-centered approaches (Box 1).

Due to the inclusion of systematic reviews with a focus on disparate outcomes, populations and methodological approaches, we expected, and found, a high degree of heterogeneity across these. However, there were some common threads across some of the reviews. These were particularly obvious when the reviews’ aims were similar (for example, the four systematic reviews on person-centered trajectories of perinatal depressive symptoms^14,28,34,35^), but commonalities also arose across systematic reviews focused on different populations and/or methodological approaches. For instance, Danielsson and colleagues^42^ found that trajectories of mental-health symptoms in children with neurodevelopmental disabilities mirrored those in typically developing children, in line with evidence reviewed in ref.^44^ and ref.^30^. Many systematic reviews found common factors associated with different trajectories, with girls/women and people in comparatively disadvantaged socioeconomic positions typically having worse trajectories than boys/men and people in comparatively advantaged socioeconomic positions, and people with social support typically having better trajectories than those without it. Another commonality across most reviews focused on person-centered approaches was the finding of a stable trajectory with a low level of symptomatology (or high level of wellbeing) as the most prevalent one. Crucially, however, this relatively consistent finding may be, as highlighted by Infurna and colleagues^19^, at least partly due to the statistical assumptions made, knowingly or not, when implementing these person-centered approaches in the primary studies—chiefly, that the different identified trajectories have the same variance or that the rates of change within each of the trajectory groups are the same over time. As a result, these models can overestimate the prevalence of certain trajectories, including stable, low-symptom trajectories, if those assumptions are not met^19^. This contrasts with variable-centered approaches, where such inconsistencies may be easier to spot. Because variable-centered models are applied to pre-defined, ‘observed’ groups, a direct comparison between descriptive data and model-implied trajectories can more easily reveal discrepancies. In person-centered analyses, the groups themselves are ‘unobserved’ products of the model, making such validation less direct. To enhance the validity of findings from either approach, a principled and transparent methodology is crucial, including the practice of visualizing observed individual trajectories against model-implied ones, as recommended by the GRoLTS checklist^50^.

The idea of relationality, interdependency and linked lives^51^ was apparent across many of the included reviews. As mentioned above, social support was identified across multiple reviews as a key protective factor, one that, crucially, can be a concrete intervention. Some of the included reviews were focused on the impact of the lives of others on an individual’s mental-health trajectories^22,28,32,33,35^. These different reviews showcase how trajectories of psychological capacities are interrelated across generations for multiple individuals across their life courses, and how changes in one person’s capacities (across different intrinsic capacity subdomains) can lead to declines in another person’s mental-health outcomes and vice versa. This highlights the idea that optimal development and healthy aging is a process that starts with birth, if not before, in line with the WHO healthy aging and life-course frameworks^2,3^.

There was heterogeneity within the included systematic reviews. Some of the key factors that may underlie this were the use of different sampling designs, measurement tools and populations of interest, as these were quite varied in some cases. This also applied to systematic reviews that were concerned with the potential impact of specific events or life transitions (for example, COVID-19, natural disasters and so on). This may also be driven by the fact that most reviews covered different populations, at different life stages, and where the measurement tools assessing psychological capacity and mental-health problems were often different and generally relied on self-reported measures.

Nevertheless, most reviews focused specifically on common mental disorders and trauma-related disorders, with very few reviews assessing eating disorders and/or psychosis. This could reflect the lack of primary studies in these areas, or gaps in evidence synthesis.

Although this umbrella review successfully synthesized a broad body of evidence, the methodological quality of the included systematic reviews, as assessed by the AMSTAR-2 tool, was highly variable. Perhaps the most critical limitation impacting the interpretation of our findings relates to the very limited number of reviews that performed a risk-of-bias assessment and incorporated the findings from this assessment in the interpretation and discussion of results. In the context of the study of trajectories of psychological capacity, some of the key potential biases include inappropriate adjustment for variables that may be in the pathway, selection bias and missing data, and measurement bias, among others^52^. Even among those reviews that included a risk of bias assessment, we were unable to review each primary study included to assess whether specific strategies were put in place to address these and other potential biases. The implications of this limitation for our umbrella review are twofold. First, the conclusions drawn from any single systematic review should be treated with caution, given these common methodological shortfalls. Second, it suggests that the evidence base on the trajectories of psychological capacities is built on a foundation of primary studies whose own risks of bias have often been unassessed and unaccounted for. Therefore, although our synthesis provides a comprehensive map of the existing literature, its findings must be interpreted in light of these field-wide methodological limitations.

Related to this, many of the systematic reviews did not acknowledge the lack of diversity in the reviewed studies when synthesizing their evidence. As a result, they missed the opportunity to qualify the limited generalizability of findings to other contexts or to subgroups that were underrepresented, or not represented at all, within the study populations. This gap may reflect the limitations of existing data infrastructure, including longitudinal cohorts. We encourage future systematic reviews to adopt a more comprehensive approach and, where possible, include non-English-language datasets in their searches to capture a broader range of evidence.

In relation to not being able to review each primary study included, it is possible that there was overlap across reviews. Furthermore, although our search was comprehensive and followed previous search strategies that were developed to assess psychological capacity, many of the systematic reviews only included primary studies published in English. Therefore, it is possible that other primary studies may have been published in other languages. A methodological limitation of this review is that we did not formally calculate the inter-rater reliability for the data extraction and quality assessment phases. Although these processes were conducted by two independent reviewers to minimize errors and bias, a quantitative measure of agreement is not available.

Two more limitations apply to the two main concepts of the review—‘trajectory’ and ‘psychological capacity’—as there is no complete agreement on what these terms mean. Regarding the concept of ‘trajectory’, we aimed to clarify our approach to this concept by focusing on studies including three or more repeated observations of the same outcome within the same individuals; however, some of the reviews did not provide a separate account for those studies, instead pooling them together with others that may not meet that criterion. Moreover, it is possible that we failed to identify reviews focused on trajectories (as per our operationalization) if they did not use this term, and the same applies to primary studies that may have remained undetected by reviews concerned with trajectories. In operationalizing ‘psychological capacity’ for this review, we built upon previous work^2,53–55^. We expanded the scope to include positive psychological factors^4,10,56^ alongside traditional mental-health indicators, ensuring our approach was consistent with the capacity-focused approach of the WHO’s healthy aging framework^2^ and the person-centeredness proposed by the WHO life-course framework^3^. Although our review may contribute to the operationalization of psychological capacity, further developments in its conceptualization may inform future updates to this umbrella review. Given the included systematic focus on trajectories of mental ill-health and wellbeing outcomes, our umbrella review may be of great interest to stakeholders interested in mental health.

### Implications and recommendations

Our umbrella review identified clear gaps in the literature about trajectories of psychological capacity. Most reviews focused on common mental disorders, with fewer reviews identified for positive mental-health outcomes and other mental-health problems (that is, eating disorder, psychotic and bipolar symptomatology). This may in part reflect the comparative novelty of some of the more positive concepts (for example, optimism and self-realization), as well as the large impact on the global burden of disease of some of the more studied concepts (that is, depression and anxiety). However, although counteracting and preventing mental ill-health is of paramount importance to improve population health^6^, the promotion of wellbeing is also key to fostering healthy aging from a holistic rather than deficit-oriented perspective. Evidence on the trajectories of both negative and positive outcomes can contribute to identify key turning points over the life course where support may be best placed. This, combined with further clinical studies on intervenable predictors of improvement and deterioration, could inform interventions at the population level. Groups at the early and later stages of life were under-studied across the reviews, and those few studies were specifically focused on life transitions, which are critical stages for the development of mental-health problems^57,58^, with young men often over-represented in reviews of post trauma symptomatology as they are often focused on military populations from high-income countries. There is strong evidence suggesting the importance of early-life mental health for a variety of outcomes, including subsequent physical diseases, mortality later in life^59^, and accelerated biological aging^60^, with one study suggesting that up to 14% of differences in physical and mental capacities among older adults can be explained by early life and developmental factors^61^. However, few reviews were able to truly focus on life-course trajectories, primarily because of lack of data. Historical birth cohorts are not common in many countries, and although there are some primary studies focusing on life-course trajectories (for example, refs. ^62–65^), these are exceptions. The comparative lack of reviews focusing or extending into older adulthood is also concerning, particularly as the number of older people is increasing globally, and in this stage of life people can experience several different life transitions, including retirement, widowhood, an increase in the number of chronic conditions, living with frailty or dementia, and so on, which have all been associated with a decline or increase in mental-health problems^66–69^. It was also noteworthy that most reviews did not address whether the primary studies included individuals undergoing treatment for their mental-health conditions, nor did they consider how treatment might influence the distribution and shape of those trajectories. Although this was not the main aim of this review of reviews, we hope future reviews will address that aspect.

Many reviews focused on social inequalities, with socioeconomic indicators, sex/gender and ethnicity being frequently explored as factors tied to different trajectories. It was less often that the reviews, even when interpreting the findings, engaged with the potential upstream, fundamental causes of those social inequalities, such as the different exposure to stigma and discrimination or the unequal and unfair distribution of and access to resources^70,71^. This may inadvertently reinforce the idea that these inequalities are, in some way, inherent to the individuals instead of socially produced, limiting the transformative impact of the findings. In line with this, a few of the included reviews^21,25,26,35^ engaged with notions of intersectionality: the idea that, due to interlocking systems of oppression, experiences of power and privilege can vary across combinations of different social identities and positions rather than simply add to one another^72,73^. Further engagement with these critical frameworks in future reviews may enhance the sensitivity to underlying inequalities as well as increase the transformative power of the synthesized evidence towards social equity to avoid leaving no one behind^2^. Nevertheless, the findings of this Analysis paper suggest that life-course interventions for psychological capacity should prioritize early identification and support for individuals at risk, including girls/women and those experiencing socioeconomic disadvantage, as these groups are more likely to have negative psychological trajectories. These findings also highlight the need for interventions addressing the mechanisms driving these inequalities and that incorporate and promote social support as a protective factor.

There was a surprising imbalance in the focus on person-centered approaches across the reviews. Although these approaches are undoubtedly helpful to characterize different trajectories within populations and may be particularly consistent with some concepts (such as resilience as a dynamic process^74^), on some occasions the rationale for exclusively focusing on heterogeneous trajectories, as identified by such methods, was unclear. Reviews of the literature on developmental or normative trajectories of psychological capacity outcomes over time were comparatively lacking. These can be very informative to summarize evidence on key vulnerability periods or inequalities across populations, and even across generations within populations, in light of recent findings of generational health drift in health outcomes^75^.

Moreover, future systematic reviews including (or focusing on) studies using person-centered approaches should engage with the risk of bias that can derive from certain methodological choices and assumptions, as highlighted by Infurna and others^19^. Although not originally devised as quality assessment tools, checklists such as the GRoLTS^50^ can be used to assess the potential risk of bias of studies using such approaches^35^. Although there may be a lack of evidence provided in the original studies to complete the checklist, its application should help qualify some of the findings in light of the abovementioned limitations and may impulse the field towards more transparent reporting practices. Based on the methodological challenges identified in this Analysis, we issue a strong recommendation for greater transparency in research that aims to document trajectories across the life course. Future systematic reviews on this topic must (1) provide a clear, operational definition of ‘trajectory’ and apply it consistently in their inclusion criteria, and (2) explicitly report the number of repeated observations for the primary studies they include. Similarly, primary studies must transparently report the distribution of their repeated observations (for example, mean, median and range) to facilitate future synthesis.

## Conclusions

Multiple systematic reviews have focused on different mental-health outcomes across different populations and the life course, with depressive, anxiety and post-traumatic symptomatology being the most frequent outcomes under study. Systematic reviews on other mental-health outcomes and psychological capacities (including, for instance, eating disorders, psychoses or subjective wellbeing) were comparatively lacking, as well as representation from people not living in high-income countries and/or in minoritized and marginalized groups within populations. Inequalities in the trajectories were reported in most of the systematic reviews. Girls and women, people in comparatively disadvantaged socioeconomic positions, and those without social support generally experienced comparatively worse trajectories across the life course, pointing at the need for preventive interventions to maximize capacity and reduce inequities. Reviews including primary studies using person-centered methods frequently reported the existence of four distinct trajectories (relatively unimpacted, recovery/improvement, chronic and delayed onset/decline). However, it is crucial to note that some of these commonalities may be due to methodological assumptions. Future reviews may engage with the quality assessment of the analytical approaches used in such studies, as well as extend the reviewed evidence to studies on trajectories using other non-person-centered approaches.

## Methods

In this umbrella review, we followed the PRISMA (Preferred Reporting Items for Systematic Reviews and Meta-Analyses) guidelines^76^ and the recommendations by the Joanna Briggs Institute (JBI) Umbrella Review Methodology Working Group^12^. A completed PRISMA checklist is included in Supplementary Table 2. The study protocol was registered in PROSPERO (CRD42023490490). We deviated from the pre-registered protocol to enhance the Review’s consistency. Specifically, we excluded any systematic reviews that did not include extractable data fitting our definition of trajectories, regardless of the terminology used by the original authors. Eligibility criteria are shown in Table 3.

**Table 3:** Eligibility criteria

Inclusion criteria	Exclusion criteria
We included studies that: 1. Were systematic or scoping reviews and/or meta-analyses. 2. Focused on assessing changes within the same individuals over time in the absence of an intervention. 3. Focused on positive and/or negative outcomes within the psychological capacity subdomain. We included any operationalization of mental-health trajectories, including depression, anxiety, trauma- and stressor-related, eating disorders or psychoses symptoms, psychological distress, positive and negative affect, happiness and life satisfaction, as well as additional outcomes related to psychological resources such as resilience, compassion, optimism, self-efficacy and locus of control. This is consistent with a previous review of the measurement of psychological capacity among older people^10^. 4. Focused on trajectories, conceptualized as repeated observations of the psychological capacity outcome/s under study across at least three time-points (Box 1). This included both person-centered and variable-centered approaches. 5. Were published in any language and focused on general or specific groups of the population (for example, people living with cancer, groups of people exposed to a particular event, and so on).	We excluded studies that: 1. Did not include a methods section and/or were not systematic or scoping reviews. 2. Did not include any one of our outcomes of interest. 3. Focused on interventions aimed at the outcome under study. 4. Only included evidence from primary studies using two time-points. 5. Did not focus or did not include extractable information on trajectories. 6. Were conference abstracts, book chapters or dissertations.

### Information sources

Searches were conducted on 11 December 2023 (first stage), with an update on 26 June 2025 (second stage). The databases MEDLINE, EMBASE, PsycINFO and the Cochrane Database of Systematic Reviews were searched, from inception of the respective databases up to the date of the search. To be able to capture ‘gray literature’ (including preprints deposited in archives such as medRxiv and PsyArXiv), we also explored Google Scholar (first 100 records) by manual searching.

### Search strategy

The search strategy included three key concepts: (1) psychological capacity (broad terms, including both positive and negative outcomes), (2) trajectories and (3) review. A full list of the terms used is provided in Supplementary Table 3.

### Selection process

All the citations found through searching the databases and additional sources were uploaded to EndNote^77^, which was used to manage the citation data and to exclude duplicate citations from the total collection of literature. These citations were also exported to Rayyan^78^, a cloud-based platform for screening citation data. Two reviewers (D.M.-A. and M.P.) independently screened all citations according to the inclusion and exclusion criteria. At the end of the screening, any discrepancies were resolved based on a discussion between the two independent reviewers, with additional input from a third reviewer (N.K.) in the first stage.

### Data-collection process and data items

We extracted data from eligible reviews using a manual data extraction form. Data were independently extracted by two reviewers working in pairs (D.M.-A./N.K. and M.P./N.K. in the first stage; D.M.-A./M.P. in the second) on the following domains: titles and objectives of the reviews, the number of databases searched, the timeframe of conducting the search process, country/geographical coverage, sample sizes, target population, number of trajectories, risk of bias/quality assessment tool used, and the mental-health outcomes reported in the reviews. All disagreements were resolved by consensus, with additional input from the third reviewer. The data extracted were recorded in an Excel spreadsheet (Supplementary Table 4).

### Study risk of bias assessment

We used the ‘Assessment of Multiple Systematic Reviews-2’ tool (AMSTAR-2)^52^ checklist for systematic reviews and research synthesis to assess the methodological quality of the studies included in this umbrella review. This checklist consists of 16 items dealing with different methodological aspects of a review article, including the appropriateness of the search strategies, the approach to synthesizing evidence, and potential sources of biases. The same pairs of reviewers (D.M.-A./N.K. or M.P./N.K. in the first stage, D.M.-A./M.P. in the second stage) independently evaluated the methodological quality of each of the included articles. In the case of disagreements, a consensus was reached between the reviewers, with additional input from the third reviewer.

### Effect measures

The measures of effect extracted were the ones reported by the eligible systematic reviews. Potential measures of effect included pooled estimates of intercept and slope(s) (Box 1), prevalence estimates of trajectories identified in each paper, and effect estimates for associations between specific factors and different trajectories (for example, odds ratios for memberships in specific trajectories among men).

### Data synthesis

Due to heterogeneity in how the outcome was operationalized, a (meta-) meta-analysis was not conducted. Results from the systematic reviews were narratively synthesized. Data were collated and described in tables, and a narrative description was developed. This synthesis focused on the different ways outcomes were operationalized across systematic reviews for psychological capacity trajectories; the life stages and geographical areas covered; the target populations; the main findings and heterogeneity within the systematic reviews. Depending on the number of systematic reviews, synthesis was conducted by broad areas of event/life transition exposure, outcome operationalization (for example, depressive symptomatology, anxiety symptomatology, PTSD) and population type. Reporting of bias assessments and certainty assessments as per the PRISMA checklist was not carried out.

### Reporting Summary

Further information on research design is available in the Nature Portfolio Reporting Summary linked to this Article.

## Supplementary information

Supplementary Information Supplementary Tables 1–3.

Reporting Summary

Supplementary Table 4 Data extraction form.

## Data Availability

Data were extracted from the identified systematic reviews as reported in the paper and supplementary material. The study protocol was deposited in PROSPERO, with reference CRD42023490490 (https://www.crd.york.ac.uk/prospero/display_record.php?RecordID=490490).
