# Supplementary material for: An umbrella review of psychological capacity and mental health trajectories across the life course
Source: Nat Ment Health. 2026 Feb 26;4(3):451–68. doi: 10.1038/s44220-026-00592-x (PMC12975514; doi:10.1038/s44220-026-00592-x)
Supplement: Supplementary file 1 — Supplementary Tables 1–3. [file 44220_2026_592_MOESM1_ESM.pdf]

nature mental health

Supplementary information

https://doi.org/10.1038/s44220-026-00592-x

An umbrella review of psychological capacity and mental health trajectories across the life course

In the format provided by the authors and unedited

Supplementary Table 1 – Studies excluded at full text screening with reasons for exclusion

Reason for exclusion	Reference
**Does not focus on outcomes of interest for this umbrella review**	Adams D, Dargue N, Paynter J. Longitudinal studies of challenging behaviours in autistic children and adults: A systematic review and meta-analysis. Clinical Psychology Review. 2023;104.
	Andersen FA, Johansen ASB, Sondergaard J, Andersen CM, Assing Hvidt E. Revisiting the trajectory of medical students' empathy, and impact of gender, specialty preferences and nationality: a systematic review. BMC medical education. 2020;20:52.
	Bevilacqua L, Hale D, Barker ED, Viner R. Conduct problems trajectories and psychosocial outcomes: a systematic review and meta-analysis. European Child and Adolescent Psychiatry. 2018;27:1239-60.
	Fontaine N, Carbonneau R, Vitaro F, Barker ED, Tremblay RE. Research review: A critical review of studies on the developmental trajectories of antisocial behavior in females. Journal of Child Psychology and Psychiatry and Allied Disciplines. 2009;50(4):363-85.
	Franke B, Michelini G, Asherson P, Banaschewski T, Bilbow A, Buitelaar JK, et al. Live fast, die young? A review on the developmental trajectories of ADHD across the lifespan. European Neuropsychopharmacology. 2018;28:1059-88.
	Garcia M, Rouchy E, Michel G. The role of Callous-Unemotional traits in delinquent and criminal trajectories: A review of longitudinal studies. Annales Medico-Psychologiques. 2019;177:819-28.
	Hall HK, Millear PM, Summers MJ, Isbel B. Longitudinal research on perspective taking in adolescence: A systematic review. Adolescent Research Review. 2021;6(2):125-50.
	Hanschmidt F, Lehnig F, Riedel-Heller SG, Kersting A. The stigma of suicide survivorship and related consequences - A systematic review. PLoS ONE. 2016;11(9):e0162688.
	Howlin P, Magiati I. Autism spectrum disorder: Outcomes in adulthood. Current Opinion in Psychiatry. 2017;30:69-76.

	Laub JH, Lauritsen JL. Violent criminal behavior over the life course: a review of the longitudinal and comparative research. Violence and victims. 1993;8(3):235-52.
	Lee JJ, Holmes L. Longitudinal trajectories of behavioral problems among children in out-of-home care: A systematic review. Children and Youth Services Review Vol 127, 2021, ArtID 106086. 2021;127.
	Leidy LE. Biological aspects of menopause: across the lifespan. Annual review of anthropology. 1994;23:231-53.
	Lu MC, Halfon N. Racial and ethnic disparities in birth outcomes: a life-course perspective. Maternal and child health journal. 2003;7(1):13-30.
	Luo Y, Weibman D, Halperin JM, Li X. A review of heterogeneity in attention deficit/hyperactivity disorder (ADHD). Frontiers in Human Neuroscience. 2019;13.
	Pender R, Fearon P, Heron J, y W. The longitudinal heterogeneity of autistic traits: A systematic review. Research in Autism Spectrum Disorders. 2020;79.
	Rodriguez LA, Castellano-Tejedor C, Blasco TB. Psychosocial adjustment and adaptation in young adults surviving pediatric cancer: A systematic review. Psicooncologia. 2024;21(1):37
	Savin-Williams RC, Cohen KM. Developmental trajectories and milestones of lesbian, gay, and bisexual young people. International Review of Psychiatry. 2015;27(5):357-66.
	Vallee M, Boissel A, Chevignard DM. Impact of Childhood Acquired Brain Injury on Siblings: A Narrative Review. Journal of Head Trauma Rehabilitation. 2022;37:E541.
	Vansoeterstede A, Cappe E, Lichtle J, Boujut E. A systematic review of longitudinal changes in school burnout among adolescents: Trajectories, predictors, and outcomes. Journal of Adolescence. 2023;95:224-47.
	Velotti P, Elison J, Garofalo C. Shame and aggression: Different trajectories and implications. Aggression and Violent Behavior. 2014;19(4):454-61.

	Webb L. Counting girls out: A review of suicide among young substance misusers and gender difference implications in the evaluation of risk. Drugs: Education, Prevention & Policy. 2009;16(2):103-26.
	Weinberger EC. Developmental trajectories of conduct problems across racial/ethnic identity and neighborhood context: A systematic review. Aggression and Violent Behavior. 2023;71.
	Westenberg JN, Tai AMY, Elsner J, Kamel MM, Wong JSH, Azar P, et al. Treatment approaches and outcome trajectories for youth with high-risk opioid use: A narrative review. Early Intervention in Psychiatry. 2022;16:207-20.
	Westfall NC, Nemeroff CB. The Preeminence of Early Life Trauma as a Risk Factor for Worsened Long-Term Health Outcomes in Women. Current Psychiatry Reports. 2015;17(11):90.
	Woods NF, Mitchell ES, Coslov N, Richardson MK. Transitioning to the menopausal transition: a scoping review of research on the late reproductive stage in reproductive aging. Menopause. 2021;28:447-66.
	Wysocki A, Butler M, Kane RL, Kane RA, Shippee T, Sainfort F. Long-Term Services and Supports for Older Adults: A Review of Home and Community-Based Services Versus Institutional Care. Journal of aging & social policy. 2015;27(3):255-79.
	Zamudio-Rodriguez A, Dartigues JF, Amieva H, Peres K. A Literature Review of Healthy Aging Trajectories through Quantitative and Qualitative Studies: A Psycho-epidemiological Approach on Community-dwelling Older Adults. The Journal of frailty & aging. 2021;10:259-71.
	Zwinkels H, Dirven L, Vissers T, Habets EJJ, vos MJ, Reijneveld JC, et al. Prevalence of changes in personality and behavior in adult glioma patients: A systematic review. Neuro-Oncology Practice. 2016;3(4):222-31.
**Does not focus on trajectories or does not include extractable information on trajectories**	Able ML, Benedek DM. Severity and Symptom Trajectory in Combat-Related PTSD: a Review of the Literature. Current Psychiatry Reports. 2019;21.
	Alaraisanen A, Heikkinen J, Kianickova Z, Miettunen J, Rasanen P, Isohanni M. Pathways leading to suicide in schizophrenia. Current Psychiatry Reviews. 2007;3(4):233-42.

	Amedu AN. Addressing trauma, post-traumatic stress disorder, and post-traumatic growth in breast cancer patients. World journal of experimental medicine. 2024;14(3):95565.
	Andersson J, Kankaanpaa R, Peltonen K, Munger AC, Korhonen L. Examining heterogeneity: A systematic review of quantitative person-centered studies on adversity, mental health, and resilience in children and young adults with refugee backgrounds. Comprehensive Psychiatry. 2024;135:152522.
	Beck K, Andreou C, Studerus E, Heitz U, Ittig S, Leanza L, Riecher-Rossler A. Clinical and functional long-term outcome of patients at clinical high risk (CHR) for psychosis without transition to psychosis: A systematic review. Schizophrenia Research. 2019;210:39-47.
	Berkman AM, Robert RS, Roth M, Askins MA. A review of psychological symptoms and post-traumatic growth among adolescent and young adult survivors of childhood cancer. Journal of health psychology. 2022;27:990-1005.
	Blendermann M, Ebalu TI, Obisie-Orlu IC, Fried EI, Hallion LS. A narrative systematic review of changes in mental health symptoms from before to during the COVID-19 pandemic. Psychological medicine. 2023:1-24.
	Brooks SK, Greenberg N. Recurrence of post-traumatic stress disorder: systematic review of definitions, prevalence and predictors. BMC Psychiatry. 2024;24(1):37.
	Buecker S, Luhmann M, Haehner P, Buhler JL, Dapp LC, Luciano EC, Orth U. The development of subjective well-being across the life span: A meta-analytic review of longitudinal studies. Psychol Bull. 2023;149(7):418-46.
	Cadwallader JS, Godart N, Chastang J, Falissard B, Huas C. Detecting eating disorder patients in a general practice setting: a systematic review of heterogeneous data on clinical outcomes and care trajectories. Eating and Weight Disorders. 2016;21(3):365-81.
	Cerda M, DiGangi J, Galea S, ro, Koenen K. Epidemiologic research on interpersonal violence and common psychiatric disorders: Where do we go from here? Depression and Anxiety. 2012;29(5):359-85.

	Ching BC, Downs J, Zhang S, Abdul Cader H, Penhallow J, Voraite E, et al. Research Review: The impact of the COVID-19 pandemic on the mental health of children and young people with pre-existing mental health and neurodevelopmental conditions–a systematic review and meta-analysis of longitudinal studies. Journal of Child Psychology and Psychiatry. 2025.
	Ciuffo G, Morais A, Landoni M, Costa R, Pinto TM, Lamela D, et al. Assessing mother's childbirth-related posttraumatic stress disorder during the first year postpartum: a systematic review. Journal of Reproductive and Infant Psychology. 2025;43(2):230
	Czempiel T, Mikolas P, Bauer M, Vogel S, Ritter P. Long-term courses of bipolar disorders. Der Nervenarzt. 2025;96(1):15
	DiMauro J, Carter S, Folk JB, Kashdan TB. A historical review of trauma-related diagnoses to reconsider the heterogeneity of PTSD. Journal of Anxiety Disorders. 2014;28(8):774-86.
	Dohm K, Redlich R, Zwitserlood P, Dannlowski U. Trajectories of major depression disorders: A systematic review of longitudinal neuroimaging findings. Australian and New Zealand Journal of Psychiatry. 2017;51:441-54.
	Erickson JM, MacPherson CF, Ameringer S, Baggott C, Linder L, Stegenga K. Symptoms and symptom clusters in adolescents receiving cancer treatment: A review of the literature. International Journal of Nursing Studies. 2013;50(6):847-69.
	Goemans A, van Geel M, Vedder P. Over three decades of longitudinal research on the development of foster children: a meta-analysis. Child abuse & neglect. 2015;42:121-34.
	Green SA, Graham BM. Symptom fluctuation over the menstrual cycle in anxiety disorders, PTSD, and OCD: a systematic review. Archives of Women's Mental Health. 2022;25:71-85.

	Guruge S, Butt H. A scoping review of mental health issues and concerns among immigrant and refugee youth in Canada: Looking back, moving forward. Canadian journal of public health = Revue canadienne de sante publique. 2015;106(2):e72-e8.
	Hakamata Y, Suzuki Y, Kobashikawa H, Hori H. Neurobiology of early life adversity: A systematic review of meta-analyses towards an integrative account of its neurobiological trajectories to mental disorders. Frontiers in Neuroendocrinology. 2022;65.
	Handiso D, Belsti Y, Boyle JA, Paul E, Shawyer F, Enticott JC. A systematic review and meta-analysis of longitudinal studies on posttraumatic stress disorders in refugees and asylum seekers. International Journal of Mental Health and Addiction. 2023:No Pagination Specified.
	Hohls JK, Konig HH, Quirke E, Hajek A. Anxiety, depression and quality of life-a systematic review of evidence from longitudinal observational studies. International Journal of Environmental Research and Public Health. 2021;18.
	Hoven CW, Duarte CS, Turner JB, ell DJ. Child mental health in the aftermath of disaster: A review of PTSD studies. Mental health and disasters. 2012:218-32.
	James PB, Wardle J, Steel A, Adams J. Post-Ebola psychosocial experiences and coping mechanisms among Ebola survivors: a systematic review. Tropical Medicine and International Health. 2019;24:671-91.
	Jheng YS, Chen SY, Tang ST. Posttraumatic Growth in Cancer Patients: A Review of Systematic Reviews and Meta-analyses. Palliative Medicine. 2023;37:174.
	Jones MB, Cameron VK, Lee S, McNelis AM. Traumatic stress in parents of children with congenital heart disease: A scoping review. Cardiology in the Young. 2024;34(8):1622
	Kalman JL, Bresnahan M, Schulze TG, Susser E. Predictors of persisting psychotic like experiences in children and adolescents: A scoping review. Schizophrenia Research. 2019;209:32-9.

	Kelebie M, Fentahun S, Tadesse G, Nakie G, Medfu G, Fasil B, et al. Predictors of long-term outcome of patients with schizophrenia in Africa: systematic review and meta-analysis. BMC public health. 2025;25(1):814.
	Kneale D. An Individual Participant Data Meta-Analysis Examining Health Inequalities Facing Older Lesbian, Gay and Bisexual People Aged 50 and over in the United Kingdom. Journal of Epidemiology and Community Health. 2020;74:A60-A1.
	Kristensen P, Weisaeth L, Heir T. Bereavement and mental health after sudden and violent losses: A Review. Psychiatry. 2012;75(1):76-97.
	Kuswanto CN, Stafford L, Sharp J, Schofield P. Psychological distress, role, and identity changes in mothers following a diagnosis of cancer: A systematic review. Psycho-Oncology. 2018;27:2700-8.
	Lal P, Gupta S. Psychological Impact of COVID-19 on Children and Adolescents: A Narrative Review of Mental Health Challenges, Interventions, and Long-Term Trajectories. Cureus. 2025;17(4):e81840.
	Lambert M, Sheldrake E, Denault AA, Scratch S. Depressive Symptoms in Individuals With Persistent Postconcussion Symptoms: A Systematic Literature Review and Meta-analysis. Journal of Head Trauma Rehabilitation. 2022;37:E583.
	Lapp LK, Agbokou C, Ferreri F. PTSD in the elderly: The interaction between trauma and aging. International Psychogeriatrics. 2011;23(6):858-68.
	Lee ARYB, Yau CE, Low CE, Li J, Ho RCM, Ho CSH. Severity and Longitudinal Course of Depression, Anxiety and Post-Traumatic Stress in Paediatric and Young Adult Cancer Patients: A Systematic Review and Meta-Analysis. Journal of Clinical Medicine. 2023;12.
	Ljungman L, Cernvall M, Gronqvist H, Ljotsson B, Ljungman G, Von Essen L. Long-term positive and negative psychological late effects for parents of childhood cancer survivors: A systematic review. PLoS ONE. 2014;9(7):e103340.

	Llewellyn-Beardsley J, Rennick-Egglestone S, Callard F, Crawford P, Farkas M, Hui A, et al. Characteristics of mental health recovery narratives: Systematic review and narrative synthesis. PLoS ONE. 2019;14.
	Lowell A, Suarez-Jimenez B, Helpman L, Zhu X, Durosky A, Hilburn A, et al. 9/11-related PTSD among highly exposed populations: a systematic review 15 years after the attack. Psychol Med. 2018;48:537-53.
	McClell, J., Robinson L, Potterton R, Mountford V, Schmidt U. Symptom trajectories into eating disorders: A systematic review of longitudinal, nonclinical studies in children/adolescents. European Psychiatry. 2020;63.
	Medforth N, Boyle C. Challenges, Complexity, and Developments in Transition Services for Young People with Disabilities, Mental Health, and Long-Term Conditions: An Integrative Review. Comprehensive child and adolescent nursing. 2023;46:180-200.
	Mosher CE, Winger JG, Given BA, Helft PR, O'Neil BH. Mental health outcomes during colorectal cancer survivorship: a review of the literature. Psycho-Oncology. 2016;25(11):1261-70.
	Namisango E, Bristowe K, Allsop MJ, Murtagh FEM, Abas M, Higginson IJ, et al. Symptoms and Concerns Among Children and Young People with Life-Limiting and Life-Threatening Conditions: A Systematic Review Highlighting Meaningful Health Outcomes. Patient. 2019;12:15-55.
	Nathoo D, Willis S. Distress among locally advanced breast cancer patients from diagnosis to follow-up: A critical review of literature. Journal of Medical Imaging and Radiation Sciences. 2017;48:S19-S20.
	Oh H, Winn JG, Verdugo JL, Banada R, Zachry C, Chan G, et al. Mental health outcomes of multiracial individuals: A systematic review between the years 2017 and 2022. Journal of affective disorders. 2023;24.
	Opsomer S, De Lepeleire J, Lauwerier E, Pype P. Resilience in family caregivers of patients diagnosed with advanced cancer-unravelling the process of bouncing back from difficult experiences, a hermeneutic review. European Journal of General Practice. 2020;26:79-85.

	Paczkowski MM, Galea S. Sociodemographic characteristics of the neighborhood and depressive symptoms. Current Opinion in Psychiatry. 2010;23(4):337-41.
	Pan MF, Kuo SC, Tang ST. Systematic review of trajectories of psychological distress among cancer patients under anti-treatments. Palliative Medicine. 2018;32:93.
	Pauley E, Walsh TS. Resilience in survivors of critical illness: A scoping review of the published literature in relation to definitions, prevalence, and relationship to clinical outcomes. Journal of the Intensive Care Society. 2022;23:345-58.
	Rask CU, Duholm CS, Poulsen CM, Rimvall MK, Wright KD. Annual Research Review: Health anxiety in children and adolescents-developmental aspects and cross-generational influences. Journal of child psychology and psychiatry, and allied disciplines. 2023;1.
	Reising K, Ttofi MM, Farrington DP, Piquero AR. Depression and anxiety outcomes of offending trajectories: A systematic review of prospective longitudinal studies. Journal of Criminal Justice. 2019;62:3-15.
	Ryles F, Meyer TD, Adan-Manes J, MacMillan I, Scott J. A systematic review of the frequency and severity of manic symptoms reported in studies that compare phenomenology across children, adolescents and adults with bipolar disorders. International Journal of Bipolar Disorders. 2017;5.
	Sachs-Ericsson NJ, Rushing NC, Stanley IH, Sheffler J. In my end is my beginning: developmental trajectories of adverse childhood experiences to late-life suicide. Aging & mental health. 2016;20(2):139-65.
	Salum GA, Polanczyk GV, Miguel EC, Rohde LAP. Effects of childhood development on late-life mental disorders. Current Opinion in Psychiatry. 2010;23(6):498-503.
	Serafini G, Pardini M, Monacelli F, Orso B, Girtler N, Brugnolo A, et al. Neuroprogression as an illness trajectory in bipolar disorder: A selective review of the current literature. Brain Sciences. 2021;11:1-20.
	Shrivastava AK, Karia SB, Sonavane SS, De Sousa AA. Child sexual abuse and the development of psychiatric disorders: a neurobiological trajectory of pathogenesis. Industrial Psychiatry Journal. 2017;26(1):4-12.

	Sweeney S, MacBeth A. The effects of paternal depression on child and adolescent outcomes: A systematic review. Journal of Affective Disorders. 2016;205:44-59.
	Tay J, Widger K, Stremler R. Self-reported experiences of siblings of children with life-threatening conditions: A scoping review. Journal of Child Health Care. 2022;26:517-30.
	Travers A, McDonagh T, Elklit A. Youth Responses to School Shootings: a Review. Current Psychiatry Reports. 2018;20.
	Trompeter N, Darvariu S, Brieva-Toloza AV, Opitz MC, Rabelo-da-Ponte FD, Sharpe H, et al. The prospective relationship between anxiety symptoms and eating disorder symptoms among adolescents: a systematic review and meta-analysis of a bi-directional relationship. European Child and Adolescent Psychiatry. 2024
	Utzon-Frank N, Breinegaard N, Bertelsen M, Borritz M, Eller NH, Nordentoft M, et al. Occurrence of delayed-onset post-traumatic stress disorder: A systematic review and meta-analysis of prospective studies. Scandinavian Journal of Work, Environment and Health. 2014;40(3):215-29.
	Verhees FG, Bendau A, Unger S, Donix KL, Asselmann E, Martini J. Panic disorder during pregnancy and the first three years after delivery: a systematic review. BMC Pregnancy and Childbirth. 2025;25(1):36.
	Wals M, Verhulst F. Child and adolescent antecedents of adult mood disorders. Current Opinion in Psychiatry. 2005;18(1):15-9.
	Waugh A, Kiemle G, Slade P. What aspects of post-traumatic growth are experienced by bereaved parents? A systematic review. European Journal of Psychotraumatology Vol 9,(1), 2018, ArtID 1506230. 2018;9(1).
	Weinberg A, Kujawa A, Riesel A. Understanding trajectories to anxiety and depression: Neural responses to errors and rewards as indices of susceptibility to stressful life events. Current Directions in Psychological Science. 2022;31(2):115-23.
	White B, Doyle J, Colville S, Nicholls D, Viner RM, Christie D. Systematic review of psychological and social outcomes of adolescents undergoing bariatric surgery, and predictors of success. Clinical Obesity. 2015;5(6):312-24.

	Wilson C, Turner J, Hutchinson A. A systematic review of post traumatic growth in survivors of childhood cancer. Psycho-Oncology. 2017;26:16.
	Wysocki A, Butler M, Kane RL, Kane RA, Shippee T, Sainfort F. Long-Term Care for Older Adults: A Review of Home and Community-Based Services Versus Institutional Care. 2012.
	Yagiela LM, Carlton EF, Meert KL, Odetola FO, Cousino MK. Parent Medical Traumatic Stress and Associated Family Outcomes after Pediatric Critical Illness: A Systematic Review. Pediatric Critical Care Medicine. 2019;20:759-68.
	Zajkowska Z, Walsh A, Zonca V, Gullett N, Pedersen GA, Kieling C, et al. A systematic review of the association between biological markers and environmental stress risk factors for adolescent depression. Journal of Psychiatric Research. 2021;138:163-75.
**Conference abstracts, books and dissertations**	Agius M, Grech A. ASSESSING THE TRAJECTORY OF SCHIZOPHRENIA EFFECTIVELY IN ORDER TO TREAT EFFECTIVELY. Psychiatria Danubina. 2024;36:S40
	Allen L, Astuto J. Depression among racially, ethnically, and culturally diverse adolescents. Handbook of depression in adolescents. 2009:75-110.
	Almond P. Is postnatal depression a public health problem? A review of the literature. Archives of Women's Mental Health. 2011;14:S59.
	Coulter RWS. Detecting, explaining, and reducing substance use, mental health, and violence inequities for sexual and gender minority youth and emerging adults.DP - 2018. Dissertation Abstracts International: Section B: The Sciences and Engineering. 2018;79(1):No Pagination Specified.
	Isohanni M, Cowling D. Ageing in schizophrenia: A systematic review. Schizophrenia Research. 2014;153:S265-S6.
	Isohanni M, Isohanni I. Ageing in schizophrenia: A systematic review. European Archives of Psychiatry and Clinical Neuroscience. 2015;265(1):S75.

	Kant J, Czisch A, Keller M. Trajectories of distress among women with breast cancer and implications for health-care settings - a narrative review. Journal of Psychosomatic Research. 2021.
	Kessing L, Willer I, Andersen P, Bukh J. Developmental, temperamental and environmental risk factors for mood disorders: Unipolar vs. bipolar trajectories. Bipolar Disorders. 2018;20:34.
	Kumar S, Gopal Das CM, iyan K, Hongally C. Trajectory of attention deficit hyperactivity disorder across the life span-focus on psychological and psychiatric issues in young adulthood. Indian Journal of Psychiatry. 2015;57(5):S180-S1.
	Martin Delawalla ML, Tiwari R, Evans YN, Rhew IC, Enquobahrie DA. 182. The Impact of the COVID-19 Pandemic on Adolescent Social Media Use, Substance Use, and Depressive Symptoms: A Scoping Review. Journal of Adolescent Health. 2022;70:S95.
	Mikels JA, Reed AE, Hardy LN, Lockenhoff CE. Positive emotions across the adult life span. Handbook of positive emotions. 2014:256-71.
	Niino K, Jester D, Mausbach B. Development of Loneliness and Social Isolation following Spousal Loss: A Systematic Review of Longitudinal Studies on Widowhood. Journal of the American Geriatrics Society. 2023;71:S337.
	Nicoll J, Maratta C, Lehr A, Parshuram C. Pediatric function within one year of critical illness: Systematic review and meta-analysis. Critical Care Medicine. 2022;50:239.
	Park J. 62.1 Predicting the Development of Serious Mental Illness. Journal of the American Academy of Child and Adolescent Psychiatry. 2019;58:S88.
	Siden H, Andrews G, Freeman A, Gregoire M, Duggal S. Clinical trajectory and symptoms in progressive conditions. Canadian Journal of Neurological Sciences. 2011;38(3):S71.
	Villafranca-West SA. Subtypes of disorder course in Vietnam veterans with war-related posttraumatic stress disorder. Dissertation Abstracts International: Section B: The Sciences and Engineering. 2011;71(11):7108.

	Yoon YJ. Discrimination and depressive symptom trajectories of middle-aged and older adults with chronic diseases.DP - 2024. Dissertation Abstracts International: Section B: The Sciences and Engineering. 2024;85(2):No Pagination Specified.
	Zwinkels H, Dirven L, Vissers T, Habets EJJ, Vos MJ, Reijneveld JC, et al. Prevalence of changes in personality and behavior in adult glioma patients: A systematic review. Neuro-Oncology. 2015;17:v194.
**Does not include a methods section and/or is not a systematic or scoping review**	Alessandri G, Vecchione M, Caprara GV. Assessment of regulatory emotional self-efficacy beliefs: A review of the status of the art and some suggestions to move the field forward. Journal of Psychoeducational Assessment. 2015;33(1):24-32.
	Anastassiou-Hadjicharalambous X, Essau C. Paediatric psychosis: Diagnosis, developmental trajectories, causes and treatment. Psychosis: Causes, diagnosis and treatment. 2012:25-44.
	Bolton EE, Tankersley AP, Eisen EM, Litz BT. Adaptation to traumatic stress: Resilient traits, resources, and trajectories of outcomes. Current Psychiatry Reviews. 2015;11(3):150-9.
	Bonanno GA. Loss, Trauma, and Human Resilience: Have We Underestimated the Human Capacity to Thrive after Extremely Aversive Events? American Psychologist. 2004;59(1):20-8.
	Bonanno GA, Chen S, Bagrodia R, Galatzer-Levy IR. Resilience and Disaster: Flexible Adaptation in the Face of Uncertain Threat. Annual review of psychology. 2023;11.
	Bonanno GA, Diminich ED. Annual Research Review: Positive adjustment to adversity--trajectories of minimal-impact resilience and emergent resilience. Journal of child psychology and psychiatry, and allied disciplines. 2013;54(4):378-401.
	Bonanno GA, Mancini AD. The human capacity to thrive in the face of potential trauma. Pediatrics. 2008;121(2):369-75.

	Bonanno GA, Mancini AD. Beyond resilience and PTSD: Mapping the heterogeneity of responses to potential trauma. Psychological Trauma: Theory, Research, Practice, and Policy. 2012;4(1):74-83.
	Bonanno GA, Westphal M, Mancini AD. Resilience to loss and potential trauma. Annual Review of Clinical Psychology. 2011;7:511-35.
	Bonanno GA, Westphal M, Mancini AD. Loss, trauma, and resilience in adulthood. Annual Review of Gerontology and Geriatrics. 2012;32(1):189-210.
	Bryant RA. The Current Evidence for Acute Stress Disorder. Current Psychiatry Reports. 2018;20.
	Bryant RA. A critical review of mechanisms of adaptation to trauma: Implications for early interventions for posttraumatic stress disorder. Clinical Psychology Review. 2021;85.
	Burgard SA, Kalousova L. Effects of the great recession: Health and well-being. Annual Review of Sociology. 2015;41:181-201.
	Colman I, Ataullahjan A. Life course perspectives on the epidemiology of depression. Canadian Journal of Psychiatry. 2010;55(10):622-32.
	Crump C. An overview of adult health outcomes after preterm birth. Early Human Development. 2020;150:105187.
	Du X, Wu H, Yalikun S, Li J, Jia J, Duan T, et al. Trajectories of Chinese adolescent depression before and after COVID-19: A cross-temporal meta-analysis with segmented regression. Journal of Affective Disorders. 2025;373:333
	Duffy A, Carlson GA. Developmental approaches to child psychopathology: Longitudinal studies and implications for clinical practice. Special Issue: Developmental Approaches to Child Psychopathology: Longitudinal Studies and Implications for Clinical Practice. 2013;22(1):6-12.
	Friedman HS, Kern ML. Personality, well-being, and health*. Annual Review of Psychology. 2014;65:719-42.

	Gaspersz R, Nawijn L, Lamers F, Penninx BWJH. Patients with anxious depression: Overview of prevalence, pathophysiology and impact on course and treatment outcome. Current Opinion in Psychiatry. 2018;31:17-25.
	Jordan G. Gender differences in the developmental trajectories, risk factors and outcomes of antisocial behaviours. Gender & Behaviour. 2011;9(1):3528-42.
	Koenig JI, Walker CD, Romeo RD, Lupien SJ. Effects of stress across the lifespan. Stress. 2011;14(5):475-80.
	Lai MC. Mental health challenges faced by autistic people. Nature human behaviour. 2023;7:1620-37.
	Lillemoe J, Holmstrom SE, Sojar SH. Emergency care considerations for transgender and gender diverse youth: A review to improve health trajectories. Current Opinion in Pediatrics. 2023;35:331-6.
	Lindert J, Sancassiani F, Massa E, Nardi AE. An Overview of the International Literature on Health-Related Quality of Life, Mental Health and Psychosocial Issues in People with Cancer. Clinical Practice and Epidemiology in Mental Health. 2021;17:253-6.
	Lindert J, Schick A, Reif A, Kalisch R, Tuscher O. Resilience trajectories-examples from longitudinal studies. [German]. Nervenarzt. 2018;89:759-65.
	Link PE, Palinkas LA. Long-Term Trajectories and Service Needs for Military Families. Clinical Child and Family Psychology Review. 2013;16(4):376-93.
	Liu P, Perez-Edgar KE. Developmental pathways from early behavioral inhibition to later anxiety: An integrative review of developmental psychopathology research and translational implications. Adolescent Research Review. 2019;4(1):45-58.
	MacLeod KB, Brownlie EB. Mental health and transitions from adolescence to emerging adulthood: Developmental and diversity considerations. Canadian Journal of Community Mental Health. 2014;33(1):77-86.

	Mancini AD, Bonanno GA. Predictors and parameters of resilience to loss: Toward an individual differences model. Journal of Personality. 2009;77(6):1805-32.
	Maughan B, Collishaw S, Stringaris A. Depression in childhood and adolescence. Journal of the Canadian Academy of Child and Adolescent Psychiatry. 2013;22(1):35-40.
	McMakin DL, Alfano CA. Sleep and anxiety in late childhood and early adolescence. Current Opinion in Psychiatry. 2015;28(6):483-9.
	Mirowsky J. Depression and the sense of control: aging vectors, trajectories, and trends. Journal of health and social behavior. 2013;54(4):407-25.
	Monroe SM, Harkness KL. Major Depression and Its Recurrences: Life Course Matters. Annual Review of Clinical Psychology. 2022;18:329-57.
	Mosolov SN, Yaltonskaya PA. Primary and Secondary Negative Symptoms in Schizophrenia. Frontiers in Psychiatry. 2021;12.
	Nagin DS, Odgers CL. Group-based trajectory modeling in clinical research. Annual Review of Clinical Psychology. 2010;6:109-38.
	Nenadic I, Falkenberg I, Mehl S, Kircher T. Long-term courses in schizophrenia: A review of current results and developments. Der Nervenarzt. 2025;96(1):5
	Orth U, Robins RW. The development of self-esteem. Current Directions in Psychological Science. 2014;23(5):381-7.
	Ozer EJ, Russo I. Development and context across the lifespan: A community psychology synthesis. xxxi, 521pp. Washington, DC: American Psychological Association; US; 2017. p. 421-36.
	Papachristou E, Frangou S, Reichenberg A. Expanding conceptual frameworks: life course risk modelling for mental disorders. Psychiatry research. 2013;206(2):140-5.

	Paunova-Markova E, Alex, rova-Karamanova A, Yordanova T, Mineva K. Impact of the Covid-19 Pandemic on Adolescent Mental Health and Well-being. Central European Journal of Paediatrics. 2023;19:54-64.
	Peleg T, Shalev AY. Longitudinal studies of PTSD: Overview of findings and methods. CNS Spectrums. 2006;11(8):589-602.
	Pfefferbaum B, Jacobs AK, Griffin N, Houston JB. Children's Disaster Reactions: the Influence of Exposure and Personal Characteristics. Current Psychiatry Reports. 2015;17(7):7.
	Pine DS, Fox NA. Childhood antecedents and risk for adult mental disorders. Annual review of psychology. 2015;66:459-85.
	Prakash J, Chatterjee K, Srivastava K, Chauhan VS. First-episode psychosis: How long does it last? A review of evolution and trajectory. Industrial Psychiatry Journal. 2021;30(2):198-206.
	Purgato M, Tedeschi F, Bonetto C, de Jong J, Jordans MJD, Tol WA, Barbui C. Trajectories of psychological symptoms and resilience in conflict-affected children in low- and middle-income countries. Clinical Psychology Review. 2020;82.
	Reckziegel R, Czepielewski LS, Hasse-Sousa M, Martins DS, de Britto MJ, Lapa CO, et al. Heterogeneous trajectories in schizophrenia: insights from neurodevelopment and neuroprogression models. Brazilian Journal of Psychiatry. 2022;44:74-80.
	Robert M, Seguin M, O'Connor K. The evolution of the study of life trajectories in social sciences over the past five years: A state of the art review. Advances in Mental Health. 2010;9(2):190-205.
	Sansom-Daly UM, Wakefield CE. Distress and adjustment among adolescents and young adults with cancer: an empirical and conceptual review. Translational pediatrics. 2013;2(4):167-97.
	Smith LH, Nist MD, Fortney CA, Warren B, Harrison T, Gillespie S, et al. Using the life course health development model to address pediatric mental health disparities. Journal of child and adolescent psychiatric nursing : official publication of the Association of Child and Adolescent Psychiatric Nurses, Inc. 2024;37(1):e12452.

	Stevens JS, van Rooij SJH, Jovanovic T. Developmental contributors to trauma response: The importance of sensitive periods, early environment, and sex differences. Current Topics in Behavioral Neurosciences. 2018;38:1-22.
	Stoffers-Winterling JM, Wiegand HF, Broll J, Schafer SK, Adorjan K, Tuscher O, Lieb K. The COVID-19 pandemic in Germany: mental health trajectories, resilient and vulnerable groups. Der Nervenarzt. 2025;96(3):266
	Warner EN, Ammerman RT, Glauser TA, Pestian JP, Agasthya G, Strawn JR. Developmental epidemiology of pediatric anxiety disorders. Child and Adolescent Psychiatric Clinics of North America. 2023;32(3):511-30.
	Warner EN, Strawn JR. Risk factors for pediatric anxiety disorders. Child and Adolescent Psychiatric Clinics of North America. 2023;32(3):485-510.
	Zahn-Waxler C, Shirtcliff EA, Marceau K. Disorders of childhood and adolescence: Gender and psychopathology. Annual Review of Clinical Psychology. 2008;4:275-303.
	Zarrella I, Russolillo LA, Caviglia G, Perrella R. Continuity and discontinuity between psychopathology of childhood and adulthood: A review on retrospective and prospective studies. Research in Psychotherapy: Psychopathology, Process and Outcome. 2017;20:101-9.
	Zurek PP, Scheithauer H. Towards a more precise conceptualization of empathy: An integrative review of literature on definitions, associated functions, and developmental trajectories. International Journal of Developmental Science. 2017;11(3):57-68.
**Mainly reviews primary studies on interventions focused on the outcome of interest**	Dewar M, Paradis A, Fortin CA. Identifying Trajectories and Predictors of Response to Psychotherapy for Post-Traumatic Stress Disorder in Adults: A Systematic Review of Literature. Canadian Journal of Psychiatry. 2020;65:71-86.
	Kuhne F, Ay DS, Marschner L, Weck F. The heterogeneous course of OCD - A scoping review on the variety of definitions. Psychiatry Research. 2020;285.

Supplementary Table 2 – PRISMA checklist

Section and Topic	Item #	Checklist item	Location where item is reported
**TITLE**			
Title	1	Identify the report as a systematic review.	N/A
**ABSTRACT**			
Abstract	2	See the PRISMA 2020 for Abstracts checklist.	2
**INTRODUCTION**			
Rationale	3	Describe the rationale for the review in the context of existing knowledge.	Intro (paragraphs 1-4)
Objectives	4	Provide an explicit statement of the objective(s) or question(s) the review addresses.	Intro (p 5)
**METHODS**			
Eligibility criteria	5	Specify the inclusion and exclusion criteria for the review and how studies were grouped for the syntheses.	Methods (p1), Table 1
Information sources	6	Specify all databases, registers, websites, organisations, reference lists and other sources searched or consulted to identify studies. Specify the date when each source was last searched or consulted.	Methods (p2)
Search strategy	7	Present the full search strategies for all databases, registers and websites, including any filters and limits used.	Methods (p3), Supplementary Table 3

Section and Topic	Item #	Checklist item	Location where item is reported
Selection process	8	Specify the methods used to decide whether a study met the inclusion criteria of the review, including how many reviewers screened each record and each report retrieved, whether they worked independently, and if applicable, details of automation tools used in the process.	Methods (p4)
Data collection process	9	Specify the methods used to collect data from reports, including how many reviewers collected data from each report, whether they worked independently, any processes for obtaining or confirming data from study investigators, and if applicable, details of automation tools used in the process.	Methods (p5), Supplementary Table 4
Data items	10a	List and define all outcomes for which data were sought. Specify whether all results that were compatible with each outcome domain in each study were sought (e.g. for all measures, time points, analyses), and if not, the methods used to decide which results to collect.	Methods (p5,7), Supplementary Table 4
	10b	List and define all other variables for which data were sought (e.g. participant and intervention characteristics, funding sources). Describe any assumptions made about any missing or unclear information.	Methods (p5), Supplementary Table 4
Study risk of bias assessment	11	Specify the methods used to assess risk of bias in the included studies, including details of the tool(s) used, how many reviewers assessed each study and whether they worked independently, and if applicable, details of automation tools used in the process.	Methods (p6)
Effect measures	12	Specify for each outcome the effect measure(s) (e.g. risk ratio, mean difference) used in the synthesis or presentation of results.	Methods (p8)
Synthesis methods	13a	Describe the processes used to decide which studies were eligible for each synthesis (e.g. tabulating the study intervention characteristics and comparing against the planned groups for each synthesis (item #5)).	Methods (p8)

Section and Topic	Item #	Checklist item	Location where item is reported
	13b	Describe any methods required to prepare the data for presentation or synthesis, such as handling of missing summary statistics, or data conversions.	Methods (p8)
	13c	Describe any methods used to tabulate or visually display results of individual studies and syntheses.	Methods (p8)
	13d	Describe any methods used to synthesize results and provide a rationale for the choice(s). If meta-analysis was performed, describe the model(s), method(s) to identify the presence and extent of statistical heterogeneity, and software package(s) used.	Methods (p8)
	13e	Describe any methods used to explore possible causes of heterogeneity among study results (e.g. subgroup analysis, meta-regression).	N/A
	13f	Describe any sensitivity analyses conducted to assess robustness of the synthesized results.	N/A
Reporting bias assessment	14	Describe any methods used to assess risk of bias due to missing results in a synthesis (arising from reporting biases).	N/A
Certainty assessment	15	Describe any methods used to assess certainty (or confidence) in the body of evidence for an outcome.	N/A
**RESULTS**			
Study selection	16a	Describe the results of the search and selection process, from the number of records identified in the search to the number of studies included in the review, ideally using a flow diagram.	Results (p1), Figure 1
	16b	Cite studies that might appear to meet the inclusion criteria, but which were excluded, and explain why they were excluded.	Supp Table 1

Section and Topic	Item #	Checklist item	Location where item is reported
Study characteristics	17	Cite each included study and present its characteristics.	Table 2
Risk of bias in studies	18	Present assessments of risk of bias for each included study.	Results (p3), Table 3
Results of individual studies	19	For all outcomes, present, for each study: (a) summary statistics for each group (where appropriate) and (b) an effect estimate and its precision (e.g. confidence/credible interval), ideally using structured tables or plots.	Supplementary Table 4
Results of syntheses	20a	For each synthesis, briefly summarise the characteristics and risk of bias among contributing studies.	Results (p3)
	20b	Present results of all statistical syntheses conducted. If meta-analysis was done, present for each the summary estimate and its precision (e.g. confidence/credible interval) and measures of statistical heterogeneity. If comparing groups, describe the direction of the effect.	N/A
	20c	Present results of all investigations of possible causes of heterogeneity among study results.	Results (p4-p20),
	20d	Present results of all sensitivity analyses conducted to assess the robustness of the synthesized results.	N/A
Reporting biases	21	Present assessments of risk of bias due to missing results (arising from reporting biases) for each synthesis assessed.	N/A
Certainty of evidence	22	Present assessments of certainty (or confidence) in the body of evidence for each outcome assessed.	N/A
**DISCUSSION**			
Discussion	23a	Provide a general interpretation of the results in the context of other evidence.	Discussion (p1-p6)

Section and Topic	Item #	Checklist item	Location where item is reported
	23b	Discuss any limitations of the evidence included in the review.	Discussion (p4-p6)
	23c	Discuss any limitations of the review processes used.	Discussion (p4-p6)
	23d	Discuss implications of the results for practice, policy, and future research.	Discussion (p8-p9)
**OTHER INFORMATION**			
Registration and protocol	24a	Provide registration information for the review, including register name and registration number, or state that the review was not registered.	Methods (p1)
	24b	Indicate where the review protocol can be accessed, or state that a protocol was not prepared.	Methods (p1)
	24c	Describe and explain any amendments to information provided at registration or in the protocol.	Methods (p1)
Support	25	Describe sources of financial or non-financial support for the review, and the role of the funders or sponsors in the review.	Acknowledgements
Competing interests	26	Declare any competing interests of review authors.	Acknowledgements
Availability of data, code and other materials	27	Report which of the following are publicly available and where they can be found: template data collection forms; data extracted from included studies; data used for all analyses; analytic code; any other materials used in the review.	Supplementary material

Note. From Page, McKenzie [14].

Supplementary Table 3 – List of terms used in OVID search (including EMBASE, MEDLINE and PsycINFO)

1	mental.mp.
2	psych*.mp.
3	depress*.mp.
4	bipolar.mp.
5	anxi*.mp.
6	wellbeing.mp.
7	well-being.mp.
8	ptsd.mp.
9	trauma*.mp.
10	distress*.mp.
11	eating disorder*.mp.
12	swb.mp.
13	happ*.mp.
14	emotion*.mp.
15	positive affect*.mp.
16	negative affect*.mp.
17	mood.mp.
18	eudemon*.mp.
19	eudaimon*.mp.
20	life satisfaction.mp.
21	satisfaction with life.mp.
22	hope.mp.
23	optimism.mp.
24	resilience.mp.
25	self-efficacy.mp.
26	humor.mp.
27	humour.mp.
28	coping.mp.
29	locus of control.mp.
30	compassion*.mp.
31	self-realisation.mp.
32	self-realization.mp.
33	agency.mp.
34	gratitude.mp.
35	1 or 2 or 3 or 4 or 5 or 6 or 7 or 8 or 9 or 10 or 11 or 12 or 13 or 14 or 15 or 16 or 17 or 18 or 19 or 20 or 21 or 22 or 23 or 24 or 25 or 26 or 27 or 28 or 29 or 30 or 31 or 32 or 33 or 34
36	trajector*.mp.
37	growth curve.mp.
38	growth model*.mp.
39	growth mixture model*.mp.
40	growth analys*.mp.
41	36 or 37 or 38 or 39 or 40
42	review.mp.
43	35 and 41 and 42

Supplementary Table 4 – Data extraction form

Please, refer to separate supplementary file.
