# Supplementary material for: An umbrella review of psychological capacity and mental health trajectories across the life course
Source: Nat Ment Health. 2026 Feb 26;4(3):451–68. doi: 10.1038/s44220-026-00592-x (PMC12975514; doi:10.1038/s44220-026-00592-x)
Supplement: Supplementary file 2 — Reporting Summary [file 44220_2026_592_MOESM2_ESM.pdf]

Corresponding author(s): Dr Darío Moreno-Agostino

Last updated by author(s): Dec 22, 2025

Reporting Summary

Nature Portfolio wishes to improve the reproducibility of the work that we publish. This form provides structure for consistency and transparency in reporting. For further information on Nature Portfolio policies, see our Editorial Policies and the Editorial Policy Checklist.

Statistics

For all statistical analyses, confirm that the following items are present in the figure legend, table legend, main text, or Methods section.

n/a	Confirmed	
☒	☐	The exact sample size (*n*) for each experimental group/condition, given as a discrete number and unit of measurement
☒	☐	A statement on whether measurements were taken from distinct samples or whether the same sample was measured repeatedly
☒	☐	The statistical test(s) used AND whether they are one- or two-sided<br>*Only common tests should be described solely by name; describe more complex techniques in the Methods section.*
☒	☐	A description of all covariates tested
☒	☐	A description of any assumptions or corrections, such as tests of normality and adjustment for multiple comparisons
☒	☐	A full description of the statistical parameters including central tendency (e.g. means) or other basic estimates (e.g. regression coefficient) AND variation (e.g. standard deviation) or associated estimates of uncertainty (e.g. confidence intervals)
☒	☐	For null hypothesis testing, the test statistic (e.g. *F*, *t*, *r*) with confidence intervals, effect sizes, degrees of freedom and *P* value noted<br>*Give P values as exact values whenever suitable.*
☒	☐	For Bayesian analysis, information on the choice of priors and Markov chain Monte Carlo settings
☒	☐	For hierarchical and complex designs, identification of the appropriate level for tests and full reporting of outcomes
☒	☐	Estimates of effect sizes (e.g. Cohen's *d*, Pearson's *r*), indicating how they were calculated

Our web collection on statistics for biologists contains articles on many of the points above.

Software and code

Policy information about availability of computer code

Data collection	No software was used for data collection.
Data analysis	EndNote version 21 was used to manage citation data and exclude duplicate citations. Rayyan (cloud-based platform) was used for screening citation data.

For manuscripts utilizing custom algorithms or software that are central to the research but not yet described in published literature, software must be made available to editors and reviewers. We strongly encourage code deposition in a community repository (e.g. GitHub). See the Nature Portfolio guidelines for submitting code & software for further information.

Data

Policy information about availability of data

All manuscripts must include a data availability statement. This statement should provide the following information, where applicable:

- Accession codes, unique identifiers, or web links for publicly available datasets
- A description of any restrictions on data availability
- For clinical datasets or third party data, please ensure that the statement adheres to our policy

A search was conducted in the databases MEDLINE, EMBASE, PsycINFO via OVID, as well as the Cochrane Database of Systematic Reviews and Google Scholar from inception until 11 December 2023. Data were extracted from the identified systematic reviews as reported in the manuscript and supplementary material. The study protocol was deposited in PROSPERO, with reference CRD42023490490.

Research involving human participants, their data, or biological material

Policy information about studies with human participants or human data. See also policy information about sex, gender (identity/presentation), and sexual orientation and race, ethnicity and racism.

Reporting on sex and gender	We report on sex/gender inequalities wherever these are reported in the included systematic reviews, following the nomenclature used by the authors.
Reporting on race, ethnicity, or other socially relevant groupings	We report on race, ethnicity, and other socially relevant groupings wherever these are reported in the included systematic reviews.
Population characteristics	No population characteristic was either an inclusion or exclusion criterion. No systematic reviews were excluded based on any population characteristic. Only systematic reviews focused on interventions aimed at the outcome under study were excluded.
Recruitment	As an umbrella review or review of systematic reviews, no new participants were recruited.
Ethics oversight	N/A

Note that full information on the approval of the study protocol must also be provided in the manuscript.

Field-specific reporting

Please select the one below that is the best fit for your research. If you are not sure, read the appropriate sections before making your selection.

☐ Life sciences ☒ Behavioural & social sciences ☐ Ecological, evolutionary & environmental sciences

For a reference copy of the document with all sections, see nature.com/documents/nr-reporting-summary-flat.pdf

Behavioural & social sciences study design

All studies must disclose on these points even when the disclosure is negative.

Study description	This study follows an umbrella review approach where systematic reviews (with or without meta-analyses) on trajectories of different psychological capacity outcomes across the life course were reviewed.
Research sample	All systematic reviews published up to the date of the last search (26 June 2025) and indexed in MEDLINE, EMBASE, PsycINFO, or the Cochrane Database of Systematic Reviews. "Grey literature" was also explored via Google Scholar.
Sampling strategy	The search strategy included three key concepts: 1) psychological capacity (broad terms, including both positive and negative outcomes), 2) trajectories, and 3) review. A full list of the terms used can be found in Supplementary Material 2. All searches were conducted on 11 December 2023. The databases MEDLINE, EMBASE, PsycINFO, and Cochrane Database of Systematic Reviews were searched, since inception of the respective databases up to the date of the search. To be able to capture 'grey literature' (including pre-prints deposited in archives such as medRxiv and PsyArXiv), we also explored Google Scholar (first 100 records) by manual searching. The study protocol was registered in PROSPERO (CRD42023490490). Eligibility criteria are shown in Table 4.
Data collection	The searches yielded 13,176 potentially eligible articles. After removal of duplicates, 8,486 remained. Title and abstract screening resulted in 210 studies included for full text assessment. After full text screening, a total of 36 reviews were selected for inclusion in our review.
Timing	The searches were conducted on 11 December 2023 and 26 June 2025, including all systematic reviews published up to those dates.
Data exclusions	4690 records were identified as duplicates and excluded. 8276 records were excluded after title and abstract screening. 174 records were excluded after full-text screening, with reasons detailed in Supplementary Table 1.
Non-participation	As an umbrella review (or review of systematic reviews), no participants dropped out of the study.
Randomization	As an umbrella review (or review of systematic reviews), no participants were randomised into groups.

Reporting for specific materials, systems and methods

We require information from authors about some types of materials, experimental systems and methods used in many studies. Here, indicate whether each material, system or method listed is relevant to your study. If you are not sure if a list item applies to your research, read the appropriate section before selecting a response.

Materials & experimental systems

n/a	Involved in the study
☒	☐ Antibodies
☒	☐ Eukaryotic cell lines
☒	☐ Palaeontology and archaeology
☒	☐ Animals and other organisms
☒	☐ Clinical data
☒	☐ Dual use research of concern
☒	☐ Plants

Methods

n/a	Involved in the study
☒	☐ ChIP-seq
☒	☐ Flow cytometry
☒	☐ MRI-based neuroimaging

Plants

Seed stocks	*Report on the source of all seed stocks or other plant material used. If applicable, state the seed stock centre and catalogue number. If plant specimens were collected from the field, describe the collection location, date and sampling procedures.*
Novel plant genotypes	*Describe the methods by which all novel plant genotypes were produced. This includes those generated by transgenic approaches, gene editing, chemical/radiation-based mutagenesis and hybridization. For transgenic lines, describe the transformation method, the number of independent lines analyzed and the generation upon which experiments were performed. For gene-edited lines, describe the editor used, the endogenous sequence targeted for editing, the targeting guide RNA sequence (if applicable) and how the editor was applied.*
Authentication	*Describe any authentication procedures for each seed stock used or novel genotype generated. Describe any experiments used to assess the effect of a mutation and, where applicable, how potential secondary effects (e.g. second site T-DNA insertions, mosiacism, off-target gene editing) were examined.*
